# Unlocking spontaneous chiral resolution in silver clusters through steric and anionic control

**DOI:** 10.1039/d5sc08561f

**Published:** 2026-02-11

**Authors:** Jin Liu, Zi-Ang Nan, Qing Li, Chuan-Qi Shen, Zuo-Bei Wang, Fu-Lin Lin, Ting Chen, Lu-Yao Liu, Zhuo-Zhou Xie, Zhu Zhuo, Wei Wang, You-Gui Huang

**Affiliations:** a State Key Laboratory of Structure Chemistry, Fujian Institute of Research on the Structure of Matter, Chinese Academy of Science Fuzhou Fujian 350002 China nanziang@fjirsm.ac.cn yghuang@fjirsm.ac.cn; b Fujian Science & Technology Innovation Laboratory for Optoelectronic Information of China Fuzhou Fujian 350108 China; c Xiamen Key Laboratory of Rare Earth Photoelectric Functional Materials, Xiamen Institute of Rare Earth Materials, Haixi Institutes, Chinese Academy of Sciences Xiamen Fujian 361021 China

## Abstract

The spontaneous emergence of chirality from racemic or achiral small molecules and nanoclusters remains a fascinating yet poorly predictable phenomenon. Herein, we report a rational design strategy for achieving spontaneous chiral resolution, as demonstrated through the controlled synthesis of a series of trinuclear silver clusters based on *C*_3_-symmetric tris(2-benzimidazolylmethyl)amine and its derivatives. Our results show that complexes formed by trinuclear clusters bearing sterically demanding ligands and trifluoromethanesulfonate (OTf^−^) favor spontaneous chiral resolution, forming homochiral structures due to helical scaffolds from bidirectional intermolecular π⋯π interactions. In contrast, trinuclear silver clusters bearing ligands with less steric hindrance paired with OTf^−^ lead to social self-sorting, resulting in racemic crystals. On the other hand, only social self-sorting was observed for the pairing of the trinuclear silver clusters with methanesulfonate (OMs^−^). Remarkably, the homochiral structures can be reversibly converted to racemic forms *via* anion exchange, enabling controllable switching between chiral and centrosymmetric polymorphs. The resulting chiral crystals exhibit optical activity and second-harmonic generation (SHG) responses, underscoring their potential for nonlinear optical applications. This work demonstrates that chiral self-sorting can be directed by systematically tuning intermolecular π⋯π interactions, offering key insights for the rational design of spontaneous resolution systems.

## Abstract 2

Spontaneous chiral resolution was achieved in *C*_3_-symmetric trinuclear silver clusters by tuning ligand steric effects and anions. Anion exchange reversibly switches homochiral and racemic polymorphs, enabling control of spontaneous resolution.

## Graphical abstract

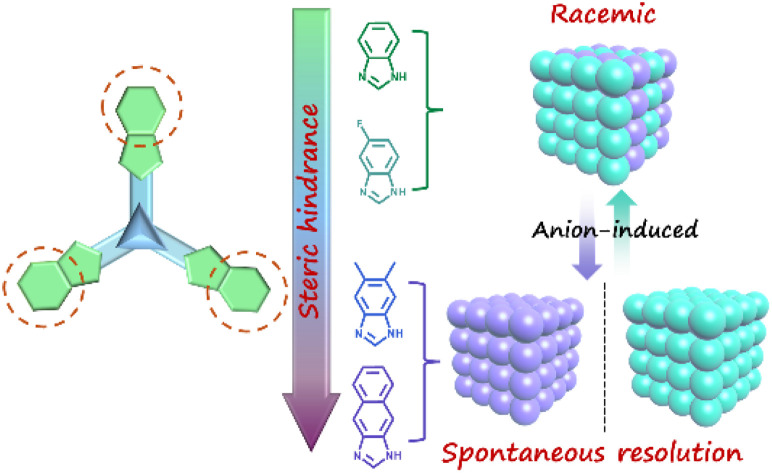

## Introduction

Chiral self-sorting refers to the spontaneous organization of racemic or achiral components into homochiral or heterochiral assemblies through chiral recognition.^1–6^ This phenomenon not only mimics the stereochemical selection in biological systems but also provides a guiding principle for designing chiral materials and functional devices. Although chirality is crucial across biology,^7–9^ asymmetric catalysis,^10–12^ and materials science,^13–16^ its spontaneous emergence from achiral systems is often considered stochastic. In most cases, obtaining enantiopure crystals still relies on the deliberate introduction of chiral auxiliaries to break the inherent symmetry.^17–20^ Although studied for decades, a universal design principle for spontaneous chiral resolution remains elusive, and the process remains largely unpredictable. In solution or soft-matter systems, chiral recognition is usually facilitated by enhancing the steric hindrance of molecular building blocks,^21,22^ for example, by the incorporation of flexible chains.^23–27^ However, this approach has been largely confined to the formation of amorphous or liquid crystalline phases, and harvesting millimeter-scale chiral single crystals *via* spontaneous resolution remains highly challenging.

Inspired by liquid-crystalline systems, a strategy involving the deliberate enlargement of molecular building blocks *via* rational organic modification has been developed to facilitate spontaneous resolution into chiral single crystals.^28–34^ In such systems, symmetry breaking during crystallization is highly dependent on specific intermolecular interactions,^35–38^ such as multiple hydrogen bonds and π⋯π stacking interactions between polycyclic aromatic groups, which are widely recognized as essential for molecular self-recognition. For instance, Yang *et al.* demonstrated that spontaneous symmetry breaking can be achieved by employing multivalent non-covalent interactions to overcome the dipole–dipole interactions that typically favor symmetric antiparallel stacking.^39^ Similarly, Jiang *et al.* reported the formation of 2D homochiral helices through orthogonal halogen and hydrogen bonding, leading to effective chiral resolution upon crystallization.^40^ Despite these advances, the strategy of deliberately manipulating steric hindrance to direct spontaneous chiral resolution remains a significantly underexplored area.

Herein, we report a systematic study on the self-assembly of a series of tripodal ligands (L^1^ = tris(2-benzimidazolylmethyl)amine, L^2^ = tris(2-fluorobenzimidazolylmethyl)amine, L^3^ = tris(2-dimethylbenzimidazolylmethyl)amine, and L^4^ = tris(2-naphthimidazolylmethyl)amine with AgOTf and AgOMs. Sterically demanding ligands (L^3^, L^4^) coordinated with AgOTf produce chiral superstructures, whereas less hindered ligands (L^1^, L^2^) form racemic crystals. Replacing AgOTf with AgOMs results in racemic products regardless of the ligand. Remarkably, anion exchange enables reversible switching between chiral and racemic polymorphs, highlighting the anion's pivotal role in defining the supramolecular outcome. The resulting chiral crystals are optically and SHG-active, imparting potential for applications in optical materials.

## Results and discussion

**Scheme 1:**
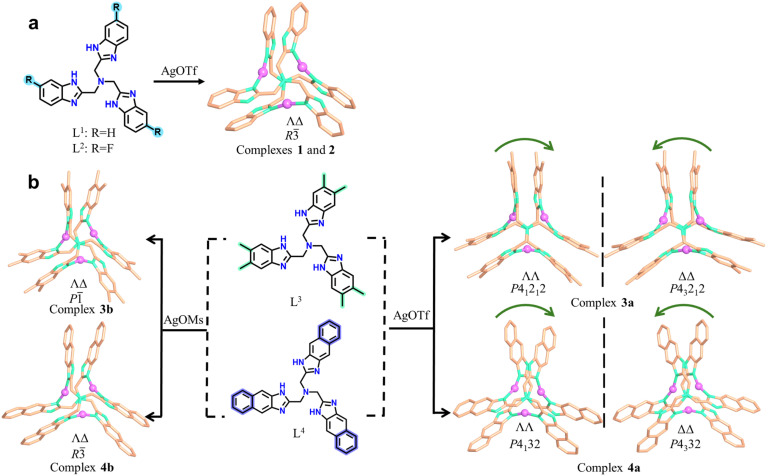
Formation of the complexes described in this study. (a) Less sterically hindered achiral *C*_3_-symmetric ligands L^1^ and L^2^ coordinate with AgOTf to form racemic crystals. (b) Sterically demanding ligands L^3^ and L^4^ coordinate with AgOMs or AgOTf to form either racemic or chiral trinuclear silver cluster complexes.

The reactions of AgOTf or AgOMs with ligands L^1^, L^2^, L^3^, or L^4^ in MeOH/acetone in the presence of tetrabutylammonium bromide at room temperature yielded crystals of different trinuclear silver clusters (Tables S1–S3). The ^1^H NMR spectra for the four ligands are shown in Fig. S1–S4.While the less sterically hindered ligands L^1^ and L^2^ resulted in racemic complexes {[Ag_3_L^1^_2_]·(OTf)_3_} (1) and {[Ag_3_L^2^_2_]·(OTf)_3_} (2), the more sterically hindered L^3^ and L^4^ produced {[Ag_3_L^3^_2_]·(OTf)_3_} (3a), {[Ag_3_L^3^_2_]·(OMs)_2_(OTf)} (3b), {[Ag_3_L^4^_2_]·(OTf)_3_} (4a), and {[Ag_3_L^4^_2_]·(OMs)_3_ (H_2_O)} (4b) (Scheme 1), among which 3a and 4a are chiral. Electrospray ionization mass spectrometry (ESI-MS) confirmed the stability of all trinuclear silver clusters in MeOH (Fig. S5–S10). These complexes are further characterized by powder X-ray diffraction (PXRD) (Fig. S11), infrared spectroscopy (IR) (Fig. S12), thermogravimetric analyses (TGA) (Fig. S13), and X-ray photoelectron spectroscopy (XPS) (Fig. S14). In the crystal structures,^41–48^ all ligands adopt either a clockwise (*Λ*) or an anticlockwise (*Δ*) conformation (Fig. S15) giving rise to two pairs of potential enantiomers of the trinuclear silver clusters: *ΛΛP*- and *ΔΔM*-[Ag_3_L_2_]^3+^, *ΛΔP*- and *ΛΔM*-[Ag_3_L_2_]^3+^ (L represents any of the four ligands of L^1^, L^2^, L^3^, and L^4^). Notably, spontaneous chiral resolution occurred during the self-assembly of 3a and 4a producing chiral single crystals. Whether spontaneous resolution takes place depends specifically on the substituents of the *C*_3_-symmetric ligands and the choice of silver salt. Therefore, we next sought to elucidate the driving force behind this symmetry-breaking phenomenon.

**Fig. 1:**
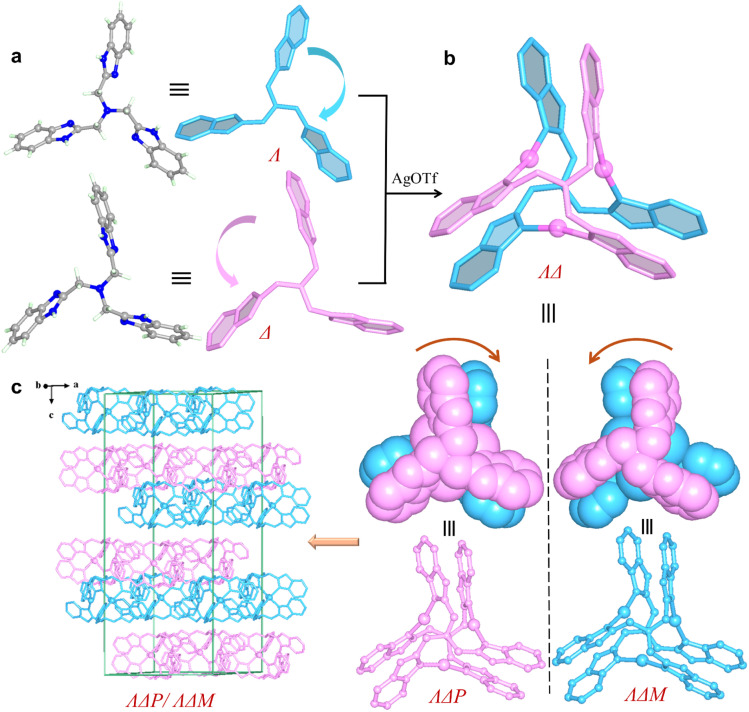
Racemic crystal structure of 1. (a) *Λ*- and *Δ*-L^1^. (b) *ΛΔP*- and *ΛΔM*-[Ag_3_L^1^_2_]^3+^. (c) *ΛΔP*-[Ag_3_L^1^_2_]^3+^ and *ΛΔM*-[Ag_3_L^1^_2_]^3+^ align alternately along the *c* axis.

Since complexes 1 and 2 are isostructural, 1 was selected for detailed crystal structure description. Single-crystal X-ray (SC-XRD) analysis reveals the centrosymmetric space group *R*3̄ for 1 (Table S1). The asymmetric unit contains one Ag^+^ ion, two one-third fragments of an L^1^ ligand, and one OTf^−^ anion. The trinuclear [Ag_3_L^1^_2_]^3+^ cluster is formed by three Ag^+^ ions in a nearly linear geometry (N–Ag–N bond angle: 167.9°) bridged by two L^1^ ligands (Fig. S16a). The two ligands adopt opposite twisting directions, clockwise (*Λ*) and anticlockwise (*Δ*), giving rise to a pair of enantiomers *ΛΔP*-[Ag_3_L^1^_2_]^3+^ and *ΛΔM*-[Ag_3_L^1^_2_]^3+^ (Fig. 1a and b). Clusters of the same handedness are linked by OTf^−^*via* hydrogen bonds, forming a chiral *α*-Po network (Fig. S16b and S17). Two such networks of opposite handedness interpenetrate with each other, leading to a racemic structure in which *ΛΔP*-[Ag_3_L^1^_2_]^3+^ and *ΛΔM*-[Ag_3_L^1^_2_]^3+^ align alternately along the *c* axis (Fig. 1c). In addition to three pairs of intramolecular π-stacked benzimidazolylmethyl arms (centroid–centroid distance of ∼3.71 Å), each [Ag_3_L^1^_2_]^3+^ also associates with a neighboring cluster of opposite handedness *via* a weak intermolecular π⋯π interaction (a centroid–centroid distance of ∼3.91 Å) (Fig. S17d).

**Fig. 2:**
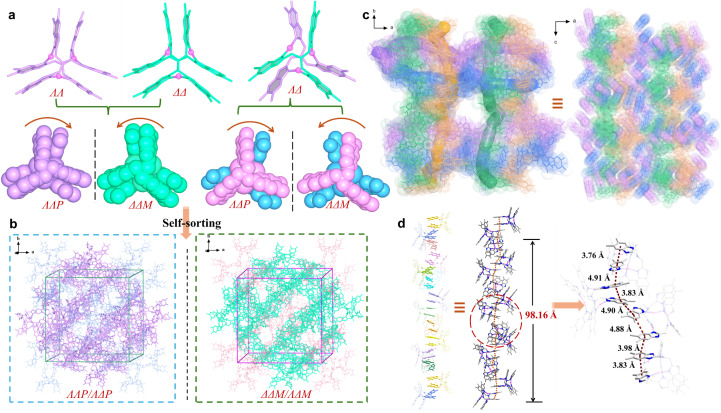
Crystal structure of 3a. (a) *ΛΛP-*, *ΔΔM*-, *ΛΔP*-, and *ΛΔM*-[Ag_3_L^3^_2_]^3+^ in 3a-*P* or 3a-*M*. (b) Narcissistic self-sorting of [Ag_3_L^3^_2_]^3+^ forming chiral structures of 3a-*P* and 3a-*M*. (c) One of the channels along the *c* axis constructed by quadruple helices in 3a-*P*. (d) One of the π-stacked helical columns with a pitch of ∼98.16 Å.

To exploit chiral single crystals, we then used more sterically hindered ligands L^3^ and L^4^ to synthesize silver clusters and successfully obtained chiral complexes 3a and 4a. The enantiomers 3a-*P* and 3a-*M* crystallize in the chiral tetragonal groups of *P*4_1_2_1_2 and *P*4_3_2_1_2, respectively (Table S2). We attempted to identify the individual enantiomer *via* macroscopic crystal morphology. Unfortunately, the morphologies of the enantiomeric crystals are nondiscernible because of exposed multiple Miller index facets (Fig. S18). Using 3a-*P* as a representative, structural analysis shows that its asymmetric unit contains four [Ag_3_L^3^_2_]^3+^clusters, two *ΛΛP*-[Ag_3_L^3^_2_]^3+^ and *ΛΔP*-[Ag_3_L^3^_2_]^3+^, resulting in an enantiomer-pure crystal structure (Fig. 2a and b). In terms of intermolecular π⋯π interactions, all [Ag_3_L^3^_2_]^3+^ clusters utilize each of their three π-stacked arms to associate bidirectionally with two neighboring clusters. As a result, each cluster associates with its six neighbors, forming a porous *α*-Po supramolecular structure with channels along the *c* axis (Fig. S19 and S21). OTf^−^ anions are located within these channels, with some engaging in NH⋯O hydrogen bonding with the host framework (Fig. S21). The over three-dimensional (3D) structure is composed of four different types of helical columns formed by dimethylbenzimidazolylmethyl arms (Fig. 2c and S20). Each helical turn comprises 24 π-stacked arms, corresponding to a pitch length of ∼98.16 Å (Fig. 2d).

**Fig. 3:**
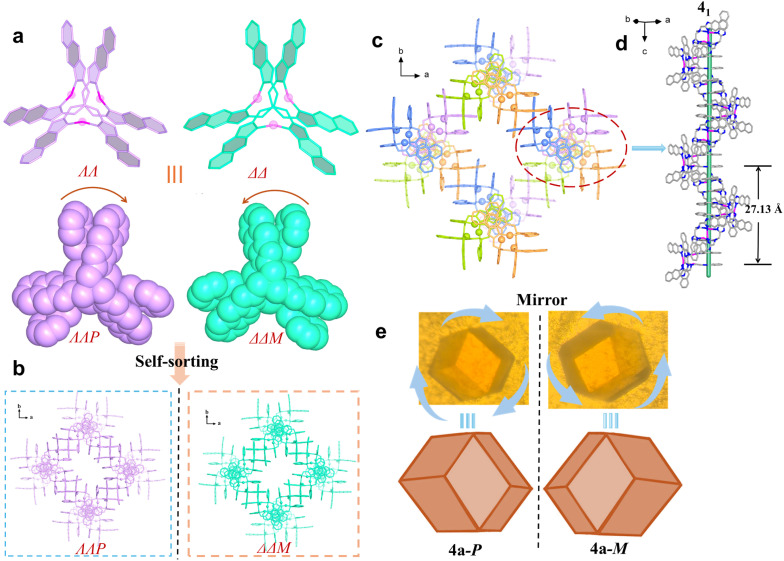
Crystal structure of 4a. (a) *ΛΛ-* and *ΔΔ*-[Ag_3_L^4^_2_]^3+^ in 4a-*P* or 4a-*M*. (b) Narcissistic self-sorting of [Ag_3_L^4^_2_]^3+^ forming chiral structures of 4a-*P* and 4a-*M*. (c) Chiral ***srs*** network of 4a-*P*. (d) 4_1_ π-stacked helical column along the *c* axis. (e) The photos of 4a-*P*/4a-*M* crystals.

The enantiomers 4a-*P* and 4a-*M* crystallizing in the cubic space groups of *P*4_1_32 and *P*4_3_32, respectively, can be clearly identified by their macroscopic crystal morphology (Fig. 3e, S22, and S23), and the structure of 4a-*P* is briefly described herein (Table S3). Its asymmetric unit contains one-third of the *ΛΛP*-[Ag_3_L^4^_2_]^3+^ cluster, and each cluster uses its three pairs of π-stacked naphthimidazolylmethyl arms to bidirectionally associate with its neighboring clusters forming a porous chiral ***srs*** network (Fig. 3 and S24). This chiral network is characteristic of two kinds of small and large helices: smaller helices composed of π-stacked naphthimidazolylmethyl arms and larger helical nanotubes formed by the helical arrangement of the silver clusters (Fig. S24). As observed in 3a, OTf^−^ anions are also entrapped within the channels as guest species, stabilized by NH⋯O hydrogen bonding interactions.

Although isostructural, the four ligands L^1^, L^2^, L^3^, and L^4^ feature distinct substitutes, and their coordination with Ag^+^ leads to chiral trinuclear Ag^+^ clusters of varying configurations. With OTf^−^, clusters bearing large steric hindrance ligands are prone to form chiral crystals *via* spontaneous chiral resolution. This process is probably due to narcissistic self-sorting, enabling homochiral clusters to assemble into π-stacked helical columns through intermolecular π⋯π interactions. The critical role of the counterion is evident, as it dictates the pathway of chiral self-sorting and the final assembled structure.

To illustrate the role of anions in spontaneous chiral resolution, crystals of chiral 3a were dissolved in methanol containing NaOMs, from which a new racemic complex 3b was harvested by crystallization. Complex 3b crystallizes in the centrosymmetric space group *P*1̄, indicating that social self-sorting occurs during the crystallization. In this crystal structure, [Ag_3_L^3^_2_]^3+^ clusters of the same handedness are triple-bridged by OTf^−^ and OMs^−^ forming homochiral columns (Fig. S25 and S26). Adjacent heterochiral columns further associate *via* intermolecular π⋯π interactions, leading to an overall two-dimensional (2D) racemic self-assembly.

Similarly, the self-assembly between L^4^ of AgOMs yields a racemic complex 4b, which is isostructural to complexes 1 and 2. In 4b, [Ag_3_L^4^_2_]^3+^ clusters of the same handedness are connected by [OMs·H_2_O]^−^ forming a chiral *α*-Po network (Fig. S27 and S28). Two such *α*-Po networks of opposite handedness interpenetrate *via* intermolecular π⋯π interactions, resulting in an overall racemic crystal structure. Social self-sorting instead of narcissistic chiral self-sorting occurred during the crystallization of complexes 3b and 4b. This difference can be rationalized by the fact that discrete [Ag_3_L^3^_2_]^3+^ or [Ag_3_L^4^_2_]^3+^ clusters are connected into chiral secondary building units (SBUs) by OMs^−^*via* hydrogen bonding. The resulting SBUs tend to undergo socially self-sorting to form racemic structures *via* intermolecular π⋯π interactions. In contrast, when OTf^−^ is the counterion, discrete [Ag_3_L^3^_2_]^3+^ or [Ag_3_L^4^_2_]^3+^ clusters directly engage in narcissistic chiral self-sorting, resulting in the homochiral structures of 3a and 4a.

**Fig. 4:**
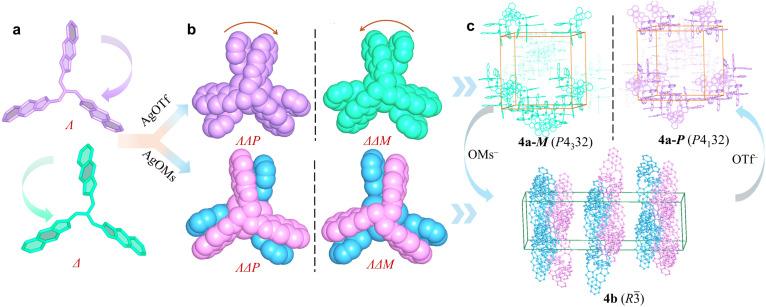
Anion-induced transition between 4a and 4b. (a) *Λ*- and *Δ*-L^4^. (b) Two pairs of enantiomers of [Ag_3_L^4^_2_]^3+^ (*ΛΛP-* and *ΔΔM*-[Ag_3_L^4^_2_]^3+^, *ΛΔP*- and *ΛΔM*-[Ag_3_L^4^_2_]^3+^). (c) Anion-induced reversible transition between 4a and 4b.

Since counterions are critical to the spontaneous chiral resolution of [Ag_3_L^4^_2_]^3+^, the reversible transformation between 4a and 4b was further studied. Dissolving crystals of 4a in methanol containing OMs^−^, crystals of 4b are obtained by crystallization. Conversely, crystals of 4a (both 4a-*M* and 4a-*P*) were produced from the solution crystals of 4b in methanol containing OTf^−^ (Fig. 4 and S29). This reversible interconversion indicates that reversible Ag^+^ coordination and dissociation occur readily in solution, allowing [Ag_3_L^4^_2_]^3+^ clusters of diverse configurations to coexist in equilibrium. However, the final self-assembly pathway, either social self-sorting or narcissistic chiral self-sorting, is decisively directed by the nature of the co-crystallizing counterion.

**Fig. 5:**
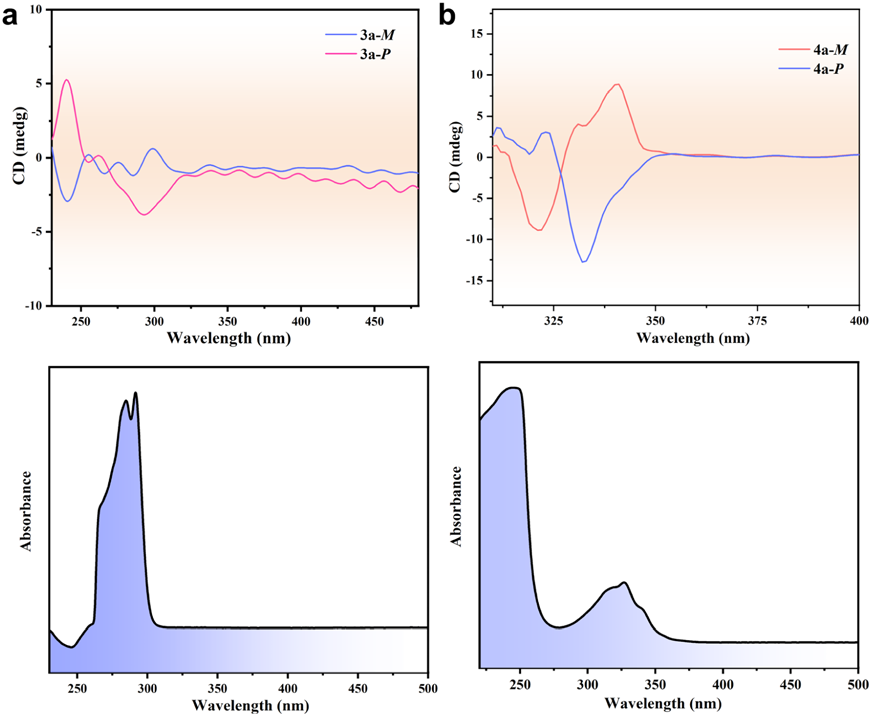
CD spectra. Circular dichroism (CD) spectra and UV-vis absorption spectra of 3a (a) and 4a (b) in methanol.

The homochirality of 3a and 4a prompted us to study their chiroptical and nonlinear optical properties. Circular dichroism (CD) spectra for 3a-*M*, 3a-*P*, 4a-*M*, and 4a-*P* were collected from solutions prepared by dissolving a single crystal in 3 mL methanol. Despite that no notable difference in ^1^H NMR was observed for the pairs of 3a/3b and 4a/4b (Fig. S30–S33), both 3a and 4a are CD active. For 3a-*M* and 3a-*P*, the spectra display a pair of CD signals confirming the enantiopurity of the [Ag_3_L^3^_2_]^3+^ clusters in the bulk single crystal. A pair of similar CD signals was observed for the solutions of 4a-*M* and 4a-*P* (Fig. 5). Furthermore, both the crystalline samples for 3a and 4a show obvious second-harmonic generation (SHG) signals, verifying their non-centrosymmetric crystal structures. The SHG efficiency of 3a is estimated to be approximately 0.47% of that of KH_2_PO_4_ (KDP), and that of 4a is estimated to be approximately 0.38% of KDP under the same experimental conditions. In contrast, the SHG signal from racemic 4b was negligible. Furthermore, the SHG responses of 4a and 4b regenerated *via* anion exchange are consistent with the original samples (Fig. S34). All these results confirm that spontaneous chiral resolution occurs during the crystallization of 3a and 4a.

## Conclusion

In conclusion, we have systematically investigated how ligand steric hindrance and the anion direct the spontaneous chiral resolution of trinuclear silver clusters based on tripodal ligands L^1^, L^2^, L^3^, and L^4^. When OTf^−^ is the counterion, sterically demanding ligands [Ag_3_L^3^_2_]^3+^ and [Ag_3_L^4^_2_]^3+^ undergo narcissistic chiral self-sorting, forming homochiral crystals, whereas the less hindered analogues [Ag_3_L^1^_2_]^3+^ and [Ag_3_L^2^_2_]^3+^ form racemic crystal structures. In contrast, the presence of OMs^−^ shifts the assembly of [Ag_3_L^3^_2_]^3+^ and [Ag_3_L^4^_2_]^3+^ toward social self-sorting, resulting in racemic crystals. Remarkably, reversible transformation between the chiral crystal structure and racemic crystal structure of [Ag_3_L^4^_2_]^3+^ was achieved *via* anion exchange. The homochirality of the resolved crystals was unambiguously confirmed by circular dichroism (CD) and second-harmonic generation (SHG) measurements.

These findings demonstrate that spontaneous chiral resolution can be rationally controlled through the combined effects of steric hindrance and anionic modulation. Compared with previous examples of chiral recognition, which generally relied on chiral auxiliaries,^17–20^ halogen-bonding interactions^40^ or specific solvent effects,^49,50^ our study achieves enantiopure crystallization through the deliberate design of ligands and careful selection of counterions. These observations can be rationalized by the role of OMs^−^ in connecting discrete clusters into chiral secondary building units (SBUs) *via* hydrogen bonding, which subsequently favors social self-sorting into racemic crystals through intermolecular π⋯π interactions. In comparison, OTf^−^ allows direct narcissistic self-sorting of discrete [Ag_3_L^3^_2_]^3+^ or [Ag_3_L^4^_2_]^3+^ clusters *via* intermolecular π⋯π interactions. This dual-control strategy demonstrates that spontaneous chiral resolution can be systematically directed through steric and anionic effects that regulate intermolecular interactions, providing an effective approach for the preparation of enantiomerically pure solid-state materials.

## Author contributions

Jin Liu synthesized the complexes, performed single-crystal X-ray diffraction studies, carried out other characterization studies, and prepared the initial manuscript draft. Qing Li and Chuan-Qi Shen collected the ESI-MS data. Zuo-Bei Wang performed the XPS data analysis. Fu-Lin Lin measured the SHG responses. Ting Chen, Lu-Yao Liu, Zhuo-Zhou Xie, Zhu Zhuo, and Wei Wang assisted with manuscript preparation. Zi-Ang Nan and You-Gui Huang supervised the project and contributed to manuscript writing. All authors discussed the results and revised the manuscript.

## Conflicts of interest

There are no conflicts to declare.

## Supplementary Material

SC-OLF-D5SC08561F-s001

SC-OLF-D5SC08561F-s002

## Data Availability

The data that support the findings of this study are available from the corresponding author upon reasonable request. CCDC 2524680–2524681, 2495911–2495914, 2217498, and 2495903 contain the supplementary crystallographic data for this paper.^41–48^ Supplementary information (SI): details of the experimental materials and characterization procedures, and figures providing additional structural, SHG data, and device performance. See DOI: https://doi.org/10.1039/d5sc08561f.
