# Supplementary material for: Unlocking spontaneous chiral resolution in silver clusters through steric and anionic control
Source: Chem Sci. 2026 Feb 11;17(10):4948–54. doi: 10.1039/d5sc08561f (PMC12910360; doi:10.1039/d5sc08561f)
Supplement: SC-017-D5SC08561F-s001 [file SC-017-D5SC08561F-s001.pdf]

Supplementary Information (SI) for Chemical Science.
This journal is © The Royal Society of Chemistry 2026

Supplementary Information

Unlocking spontaneous chiral resolution in silver clusters through steric and anionic control

Jin Liu,[abc] Zi-Ang Nan,[ab]* Qing Li,[abc] Chuan-Qi Shen,[ab] Zuo-Bei Wang,[ab] Fu-Lin Lin,[ab] Ting Chen,[ab] Lu-Yao Liu,[abc] Zhuo-Zhou Xie,[ab] Zhu Zhuo,[ab] Wei Wang,[ab] and You-Gui Huang[abc]*

[a] State Key Laboratory of Structure Chemistry, Fujian Institute of Research on the Structure of Matter, Chinese Academy of Science, Fuzhou, Fujian, 350002, China.
[b] Fujian Science & Technology Innovation Laboratory for Optoelectronic Information of China, Fuzhou, Fujian, 350108, China.
[c] Xiamen Key Laboratory of Rare Earth Photoelectric Functional Materials, Xiamen Institute of Rare Earth Materials, Haixi Institutes, Chinese Academy of Sciences, Xiamen, Fujian, 361021, China.

*Correspondence to: nanziang@fjirsm.ac.cn, yghuang@fjirsm.ac.cn

Table of Contents:

1. **Materials and Physical Measurements**
2. **Crystallography**
3. **Experimental Section**
4. **Summary Tables of Single Crystal Data**
5. **^{1}H NMR and ESI-MS Characterizations**
6. **TGA, IR, PXRD, and XPS Characterizations of Complexes 1–4b**
7. **Structures of Complexes 1–4b**
8. **^{1}H NMR and SHG Responses of Complexes 3a, 3b, 4a, and 4b**
9. **Supplementary References**

1. Materials and Physical Measurements.

All the reagents except $L^1/L^2/L^3/L^4$ were purchased from commercial sources and used without purification. The ligands $L^1/L^2/L^3/L^4$ were prepared by the previously reported methods [S1]. Thermogravimetric analyses (TGA) were carried out on a Mettler Toledo analyzer, with the temperature increasing at 10 °C/min in an argon atmosphere. Fourier transform infrared spectra (FT-IR) were recorded in the range of 400–4000 cm^{-1} using a Nicolet iS 50 FTIR instrument. Powder X-ray diffraction (PXRD) patterns were recorded using a Rigaku 2100 diffractometer with CuK_α radiation (40kV, 15mA), and data were collected in the 5–40° range at a scan rate of 10°/min. The CD spectra were measured on a Jasco-1500 spectrophotometer. NMR measurements were performed on Quantum-I Plus 600 MHz. High-resolution Electrospray Ionization Time of Flight Mass Spectrometry (ESI-TOF-MS) measurement was performed on a 1290 Infinity II-6230B system. X-ray photoelectron spectroscopy (XPS) study was performed on a ThermoFisher ESCALAB 250 Xi system and the *C*1*s* line at 284.8 eV was used as the binding energy reference. Powder SHG measurements on compounds **3a**, **4a**, **4b**, and KDP were carried out using a Spectra-Physics Spirit One 1040-8 laser system operating at 1040 nm (Yb: KGW). The SHG signal was collected through an optical setup and guided into a fiber optic bundle, which was subsequently coupled to a spectrometer and detected using a CCD detector. A powdered KDP sample was used as a standard reference to evaluate the relative second-order nonlinear optical response.

2. Crystallography.

Single-crystal X-ray data for **4a** were collected on an XtaLAB Synergy diffractometer with Mo-K_α, and the data for the other single-crystals were collected on a Bruker D8 Venture diffractometer with Mo-K_α radiation micro-focus X-ray sources. Structures were solved using a direct method and refined by the full-matrix least-squares technique on F^2 with SHELXTL 2018 program and SHELXTL 2014 program for **1–4b** [S2]. The hydrogen atoms are geometrically generated and refined using a riding model. The X-ray crystallographic coordinates for structures reported in this

article have been deposited at the Cambridge Crystallographic Data Centre (CCDC), under deposition numbers CCDC 2524680–2524681, 2495911–2495914, 2217498, and 2495903. These data can be obtained free of charge from The Cambridge Crystallographic Data Centre via www.ccdc.cam.ac.uk/data_request/cif. Detailed crystallographic data are listed in Tables S1–S3.

3. Experimental Section.

Synthesis of $[Ag^{I}_3L^1_2]\cdot(OTf)_3$ (1): L^1 (20.4 mg, 0.05 mmol), AgOTf (100 mg, 0.39 mmol) and tetrabutylammonium bromide (10 mg, 0.03 mmol) were dissolved in a mixture of MeOH and acetone (4.5 mL, v/v : 3/1). The resulting turbid solution was stirred at room temperature for 2 h. And then, the insoluble matter was removed by filtration. The filtrate was left undisturbed at room temperature for 5 days. Crystals of **1** were obtained. Yield: 60% based on L^1.

Synthesis of $[Ag^{I}_3L^2_2]\cdot(OTf)_3$ (2): 2 was prepared by the same method as that of **1** but using L^2 as the ligand. Yield: 40% based on L^2.

Synthesis of $[Ag^{I}_3L^3_2]\cdot(OTf)_3$ (3a): 3a was prepared by the same method as that of **1** but using L^3 as the ligand. Yield: 30% based on L^3.

Synthesis of $[Ag^{I}_3L^3_2]\cdot(OTf)\cdot(OMs)_2$ (3b): 3a (30 mg, 0.004 mmol) and NaOMs (40 mg, 0.34 mmol) were dissolved in a solution of MeOH (5 mL). The resulting turbid solution was stirred at room temperature for 0.5 h. And then, the insoluble matter was removed by filtration. The filtrate was left undisturbed at room temperature for 10 days. Crystals of **3b** were obtained. Yield: 40% based on **3a**.

Synthesis of $[Ag^{I}_3L^4_2]\cdot 3OTf$ (4a): 4a was prepared by the same method as that of **1** but using L^4 as the ligand. Yield: 60% based on L^4.

Synthesis of $[Ag^{I}_3L^4_2]\cdot(OMs)_3\cdot(H_2O)$ (4b): L^4 (28 mg, 0.05 mmol), AgOMs (100 mg, 0.49 mmol), and tetrabutylammonium bromide (10 mg, 0.03 mmol) were dissolved in a mixture of MeOH and acetone (4.5 mL, v/v : 3/1). The resulting turbid solution was stirred at room temperature for 2 h. And then, the insoluble matter was removed by filtration. The filtrate was left undisturbed at room temperature for 5 days. Crystals of **4b** were obtained. Yield: 40% based on L^4.

4. Summary Tables of Single Crystal Data.

Table S1. Crystallographic data for **1** and **2**.

Compounds	**1**	**2**
Formula	$C_{51}H_{42}Ag_3F_9N_{14}O_9S_3$	$C_{51}H_{36}Ag_3F_{15}N_{14}O_9S_3$
Formula weight	1585.77	1693.73
Temp. (*K*)	200	200
Crystal System	Trigonal	Trigonal
Space group	$R\bar{3}$	$R\bar{3}$
a (Å)	15.6677(5)	15.8505(3)
c (Å)	40.591(2)	40.1030(14)
V(Å^3)	8629.1(7)	8725.6(4)
Z	6	6
$\rho_{cal.}$(g cm^{-3})	1.831	1.934
μ	1.218	1.226
F(000)	4728	5016
θ *range* (°)	2.600–27.507	2.941–27.493
Reflections ($I > 2\sigma$)	3954	3192
$R_1(I > 2\sigma)$	0.0305	0.0507
$wR_2(all)$	0.0863	0.1372
GOF on F^2	1.060	1.029
CCDC#	2524680	2524681

[a] $R = \sum||F_0|-|F_c||/\sum|F_0|$

[b] $wR = |\sum w(F_0-F_c)^2/\sum w(F_0^2)^2|^{1/2}$

Table S2. Crystallographic data for **3a-*M***, **3a-*P***, and **3b**.

Compounds	**3a-*M***	**3a-*P***	**3b**
Formula	$C_{252}H_{264}Ag_{12}F_{36}N_{56}O_{36}S_{12}$	$C_{252}H_{264}Ag_{12}F_{36}N_{56}O_{36}S_{12}$	$C_{63}H_{72}Ag_{3}F_{3}N_{14}O_{9}S_{3}$
Formula weight	7016.34	7016.34	1646.13
Temp. (*K*)	100	293	200
Crystal System	Tetragonal	Tetragonal	Triclinic
Space group	$P4_32_12$	$P4_12_12$	$P\bar{1}$
a (Å)	35.0187(2)	35.2602(13)	13.5048(15)
b (Å)	35.0187(2)	35.2602(13)	15.8262(17)
c (Å)	51.3873(4)	51.837(3)	20.590(3)
α (°)	90	90	104.418(3)
β (°)	90	90	97.720(3)
γ (°)	90	90	112.870(3)
V(Å^3)	63016.7(9)	64449(6)	3793.0(8)
Z	8	8	2
$\rho_{cal.}$(g cm^{-3})	1.479	1.466	1.441
μ	7.356	0.877	0.916
F(000)	28288	28286	1672
θ *range* (°)	2.133–67.493	1.868–27.502	2.144–27.605
Reflections ($I > 2\sigma$)	41384	42682	10551
$R_1(I > 2\sigma)$	0.0882	0.0793	0.0757
$wR_2(all)$	0.2651	0.2674	0.2509
GOF on F^2	1.060	1.026	1.035
Flack parameter	0.050(9)	0.07(3)	/
CCDC#	2495911	2495912	2495913

[a] $R = \sum||F_0|-|F_c||)/\sum|F_0|$

[b] $wR = |\sum w(F_0-F_c)^2/\sum w(F_0^2)^2|^{1/2}$

Table S3. Crystallographic data for **4a-*M***, **4a-*P***, and **4b**.

Compounds	**4a-*M***	**4a-*P***	**4b**
Formula	$C_{75}H_{54}Ag_3F_9N_{14}O_{4.5}S_3$	$C_{75}H_{52.5}Ag_3F_9N_{14}O_9S_3$	$C_{75}H_{69}AgN_{14}O_{12}S_3$
Formula weight	1886.11	1884.60	1877.04
Temp. (*K*)	100	200	200
Crystal System	Cubic	Cubic	Trigonal
Space group	$P4_332$	$P4_132$	$R\bar{3}$
a (Å)	27.18680(10)	27.1290(3)	17.7824(5)
b (Å)	27.18680(10)	27.1290(3)	17.7824(5)
c (Å)	27.18680(10)	27.1290(3)	46.121(2)
α (°)	90	90	90
β (°)	90	90	90
γ (°)	90	90	120
V(Å^3)	20094.4(2)	19966.5(7)	12630.3(10)
Z	8	8	6
$\rho_{cal.}$(g cm^{-3})	1.247	1.254	1.403
μ	0.709	0.713	0.829
F(000)	7552	7540	5412
θ range (°)	1.835–27.477	2.252–27.493	2.574–27.510
Reflections ($I > 2\sigma$)	6901	6428	5067
$R_1(I > 2\sigma)$	0.0697	0.0563	0.0508
$wR_2(all)$	0.2061	0.1717	0.1508
GOF on F^2	1.090	1.054	1.055
Flack parameter	0.10(7)	0.26(6)	/
CCDC#	2495914	2217498	2495903

[a] $R = \sum||F_0|-|F_c||)/\sum|F_0|$

[b] $wR = |\sum w(F_0-F_c)^2/\sum w(F_0^2)^2|^{1/2}$

5. ^{1}H NMR and ESI-MS Characterizations.

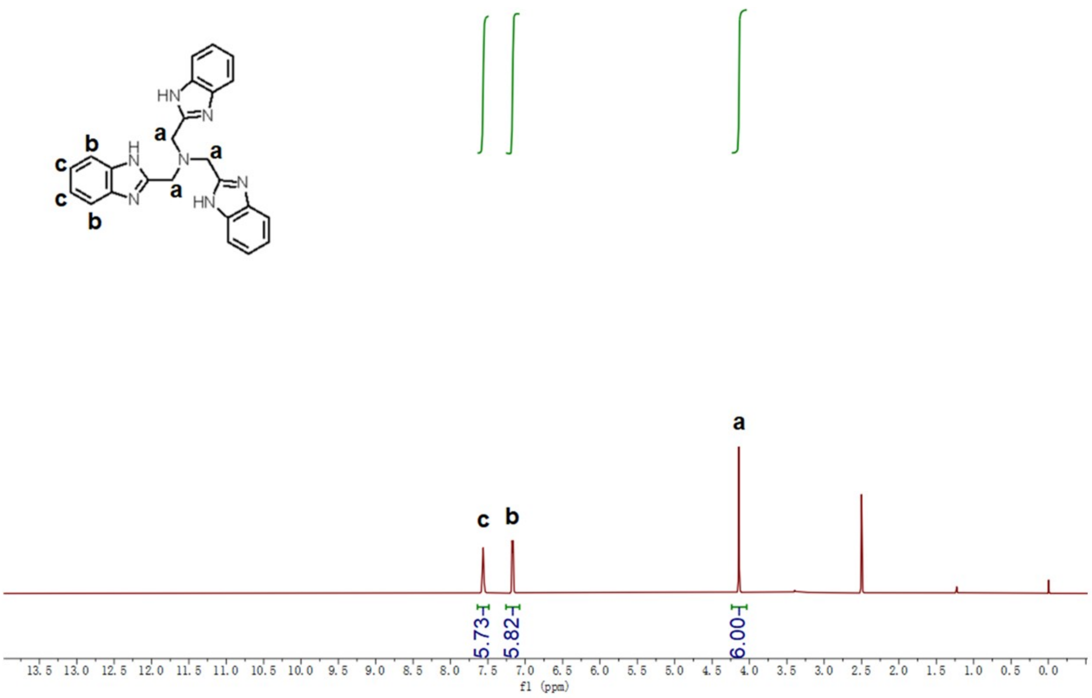

Figure S1. ^{1}H NMR of L^{1} (600 MHz, DMSO-*d6*).

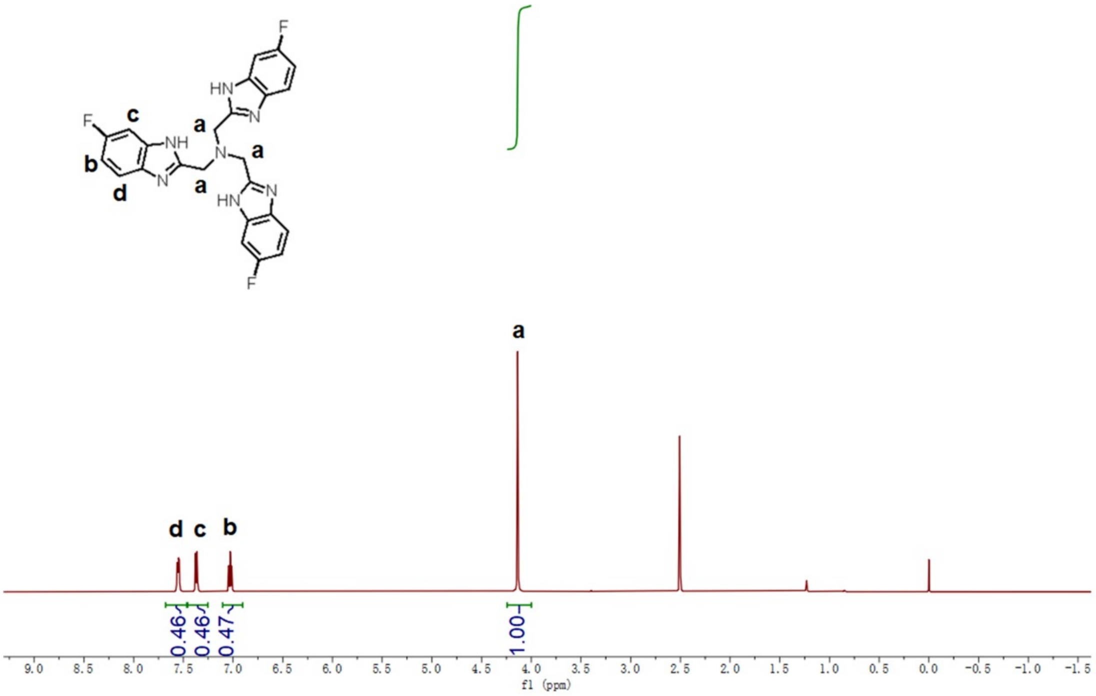

Figure S2. ^{1}H NMR of L^{2} (600 MHz, DMSO-*d6*).

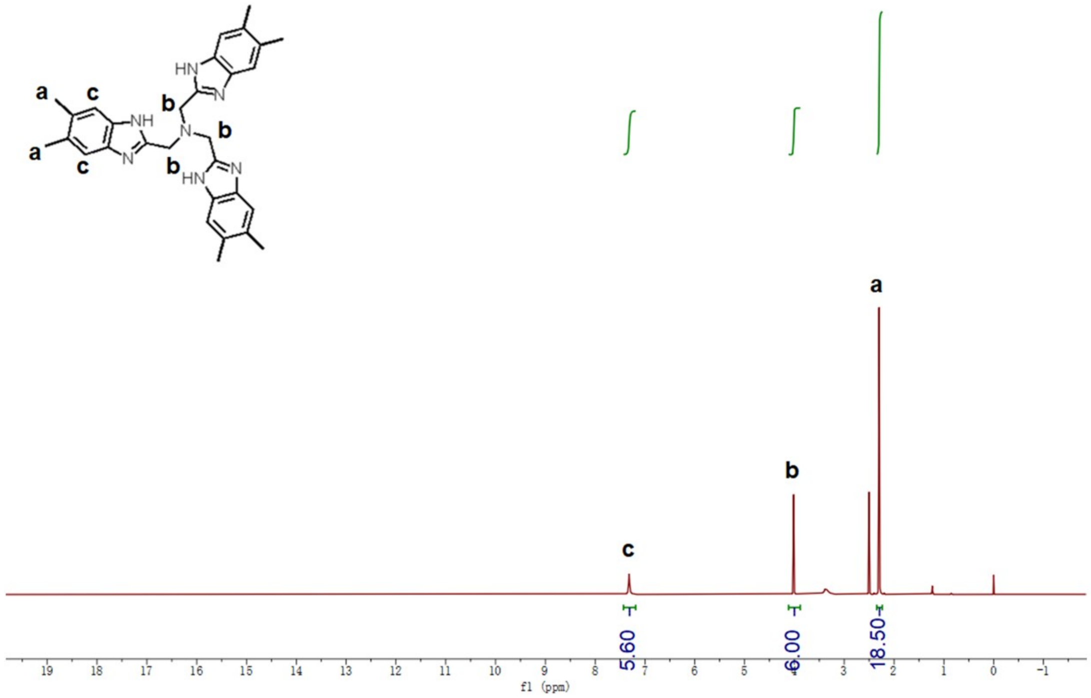

Figure S3. ^{1}H NMR of L^{3} (600 MHz, DMSO-*d6*).

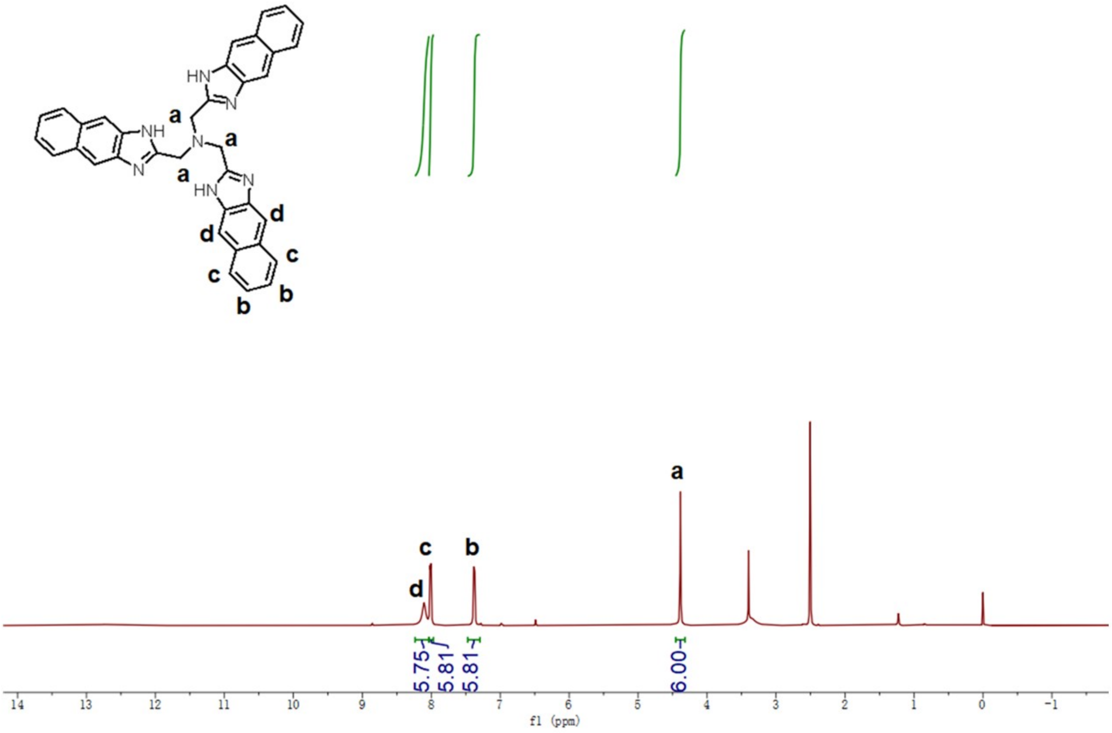

Figure S4. ^{1}H NMR of L^{4} (600 MHz, DMSO-*d6*).

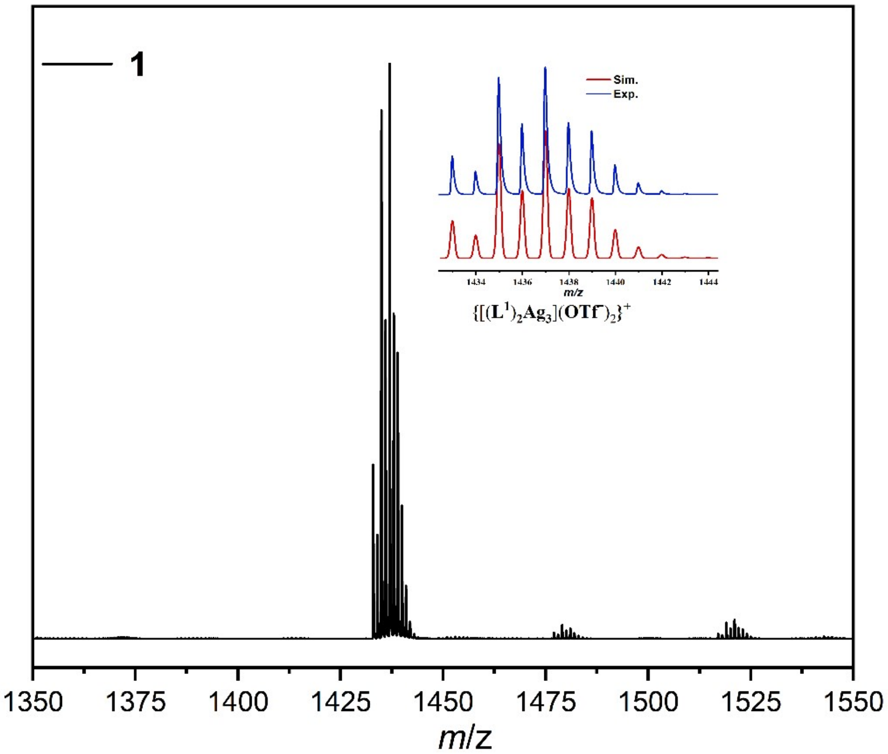

Figure S5. ESI-MS of the crystals of **1** in MeOH. Insets: Zoom-in ESI-MS of experimental (blue line) and simulated (red line) data.

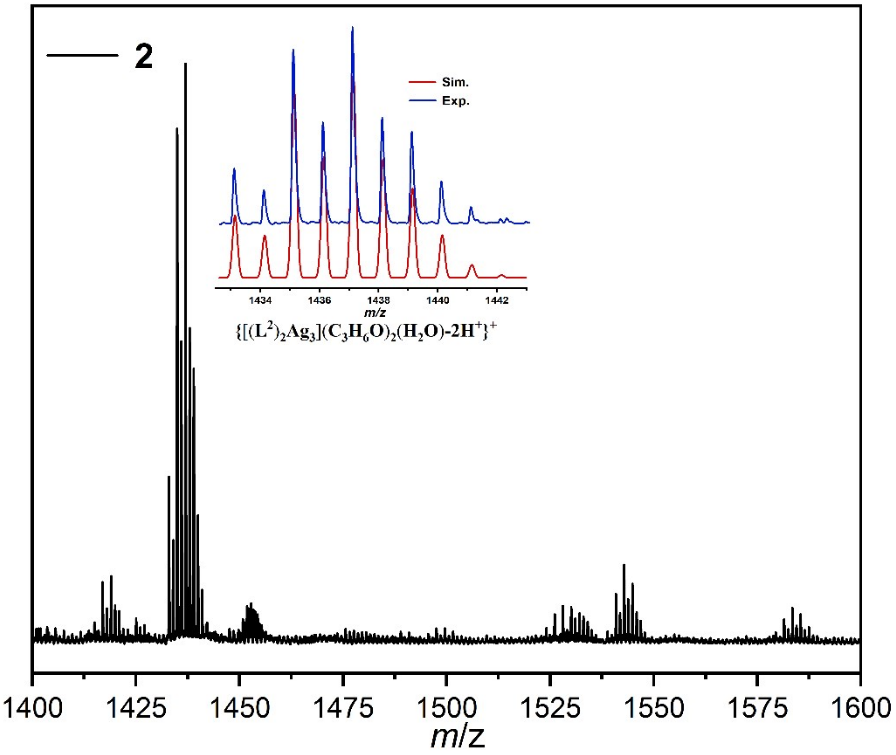

Figure S6. ESI-MS of the crystals of **2** in MeOH. Inset: Zoom-in ESI-MS of experimental (blue line) and simulated (red line) data.

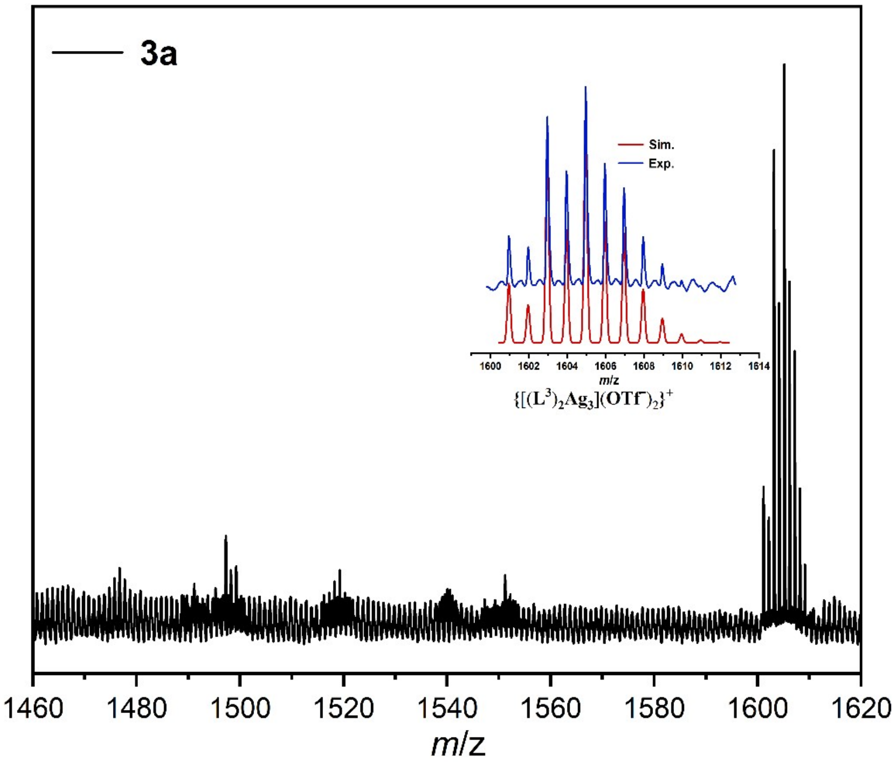

Figure S7. ESI-MS of the crystals of **3a** in MeOH. Inset: Zoom-in ESI-MS of experimental (blue line) and simulated (red line) data.

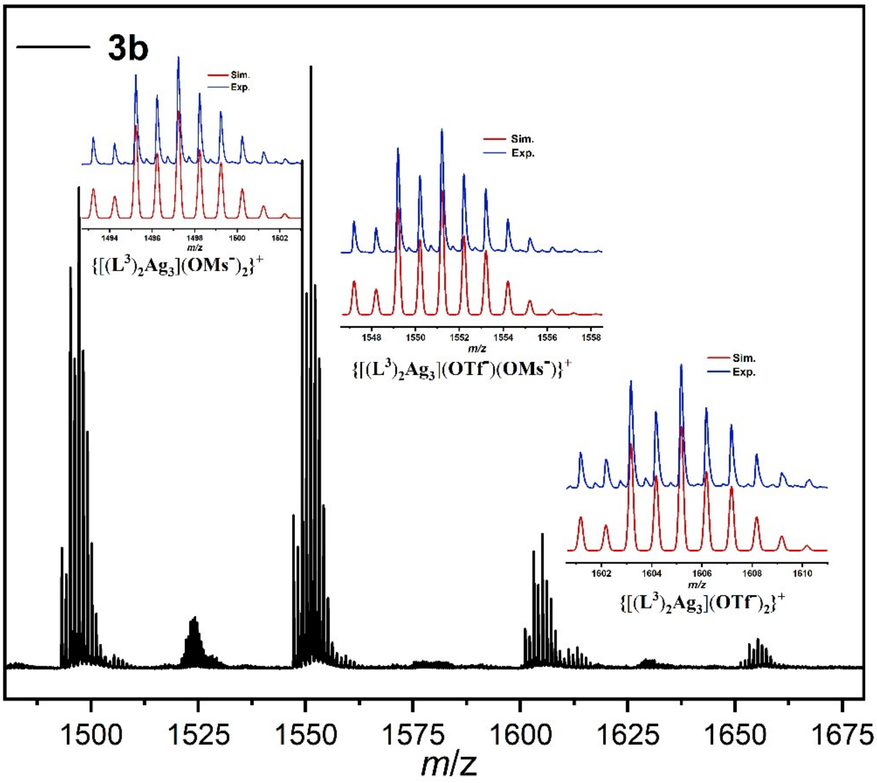

Figure S8. ESI-MS of the crystals of **3b** in MeOH. Inset: Zoom-in ESI-MS of experimental (blue line) and simulated (red line) data.

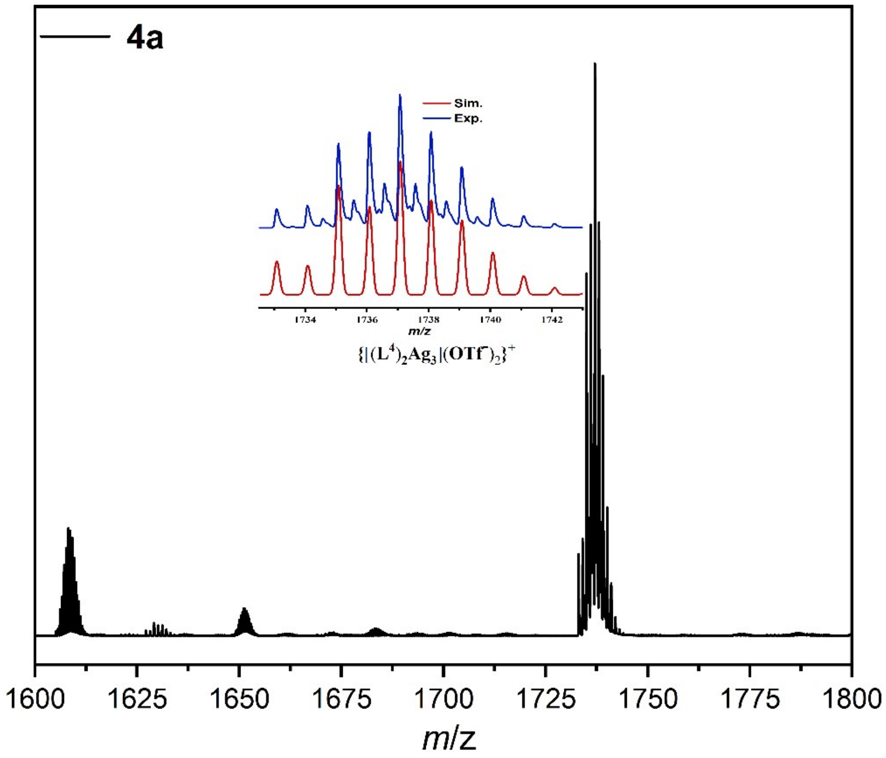

Figure S9. ESI-MS of the crystals of **4a** in MeOH. Inset: Zoom-in ESI-MS of experimental (blue line) and simulated (red line) data.

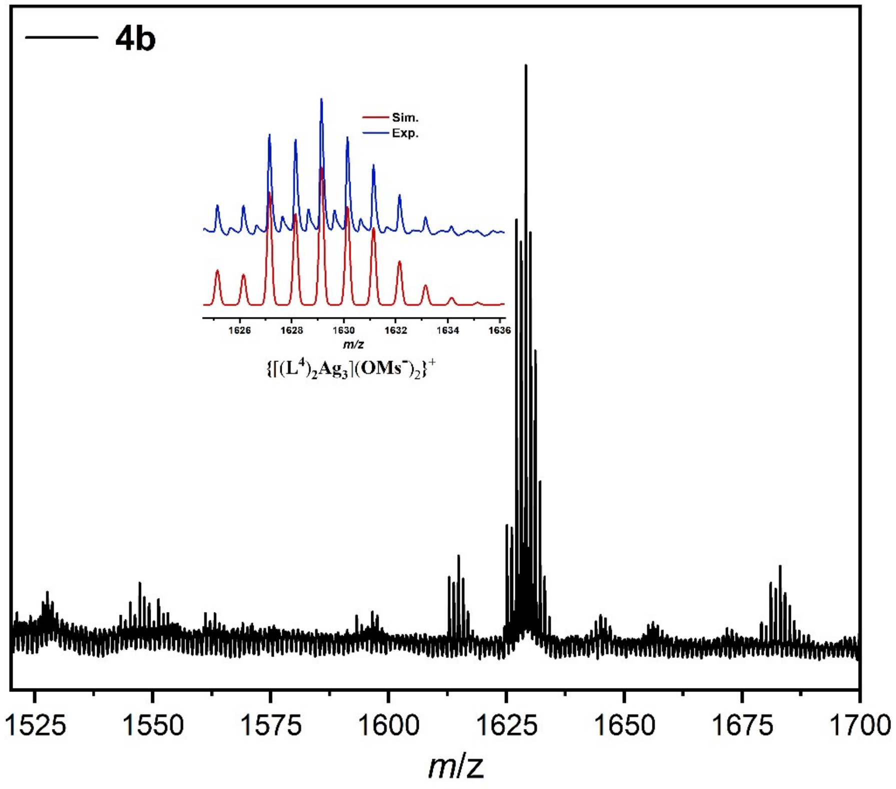

Figure S10. ESI-MS of the crystals of **4b** in MeOH. Inset: Zoom-in ESI-MS of experimental (blue line) and simulated (red line) data.

6. TGA, IR, PXRD, and XPS Characterizations of Complexes 1–4b.

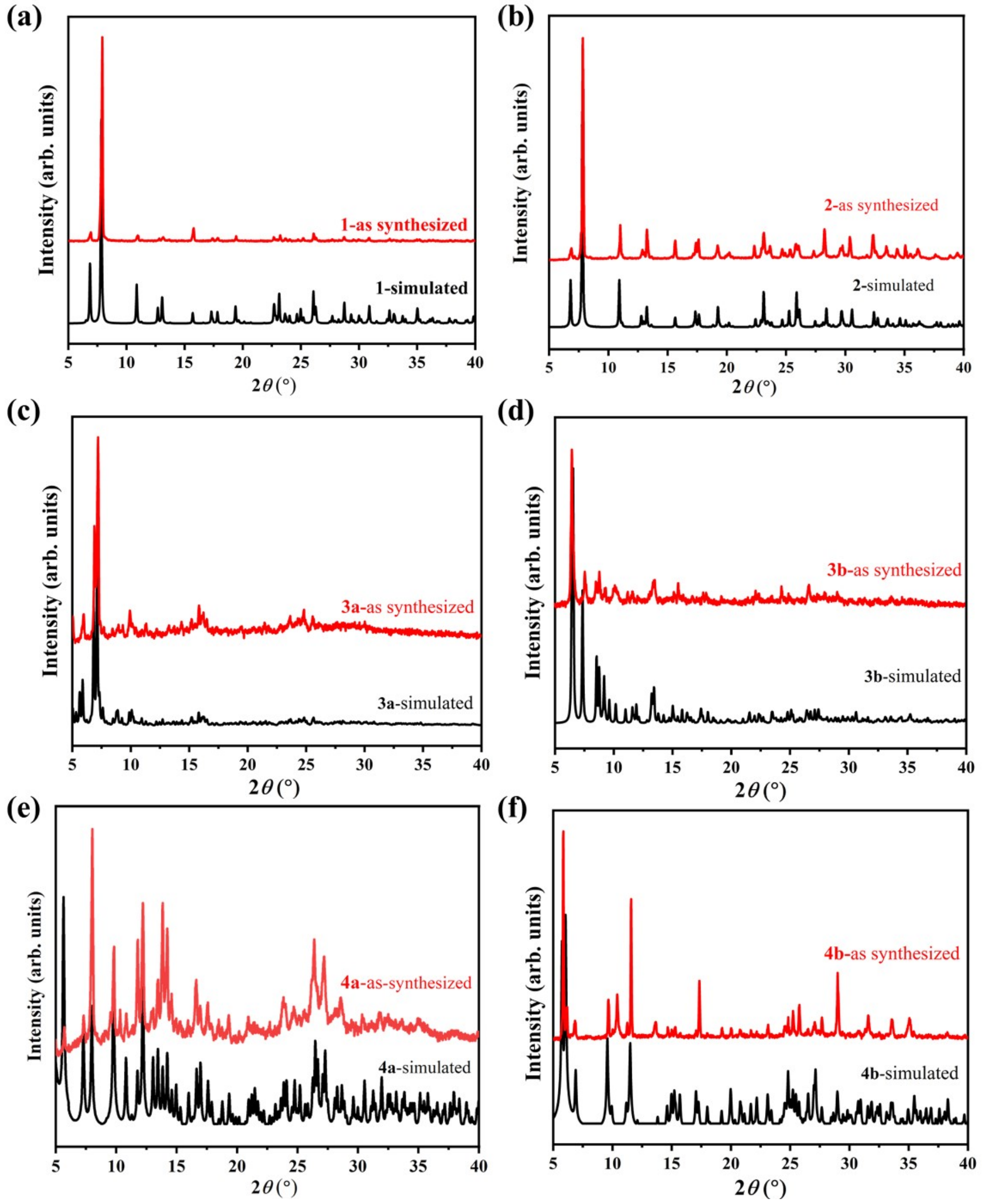

Figure S11. Simulated and experimental PXRD patterns.

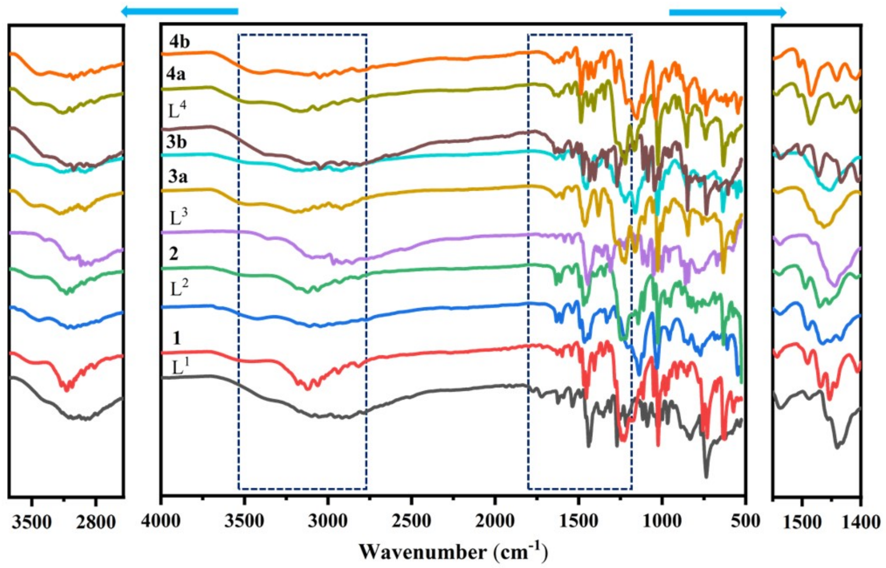

Figure S12. IR spectra of ligands L[1]–L[4] and **1–4d**. The broad band at 3700–2500 cm^{-1} can be assigned to $\upsilon_{C\text{-}H}$ of the aromatic/methylene groups and $\upsilon_{N\text{-}H}$ of the imidazole rings. The band at 1607 cm^{-1} can be attributed to aromatic $\upsilon_{C\text{-}H}$, and the band at 1446 cm^{-1} can be assigned to $\upsilon_{C=N}$ of the imidazole ring.

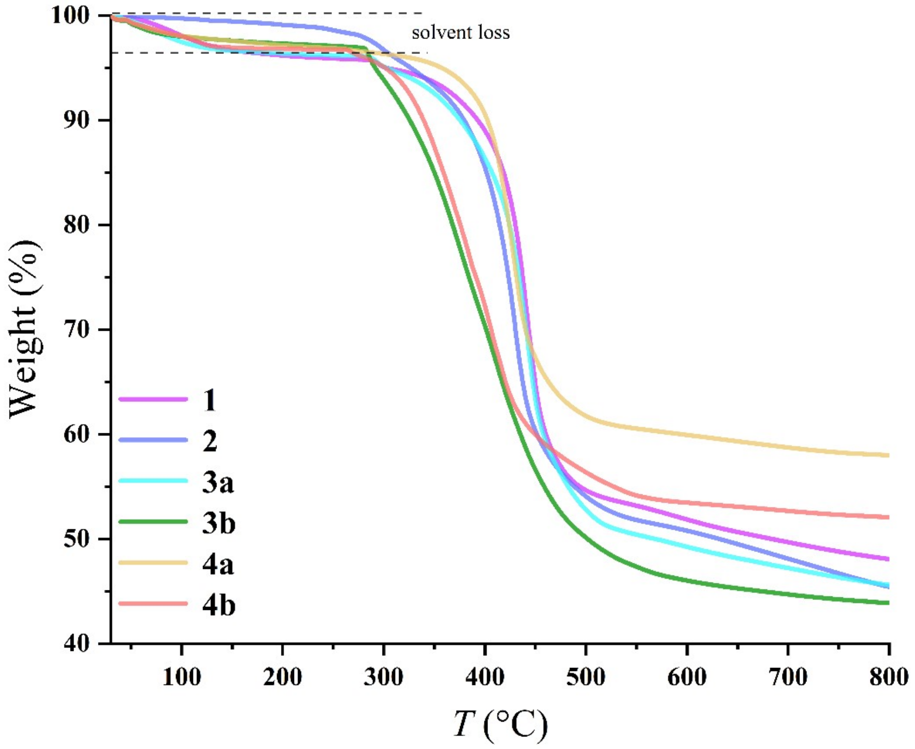

Figure S13. TGA for **1–4b**. Under an argon stream, the compounds show a weight loss of 2.1–3.5% in the range of 30–150 °C, which can be attributed to the removal of solvent.

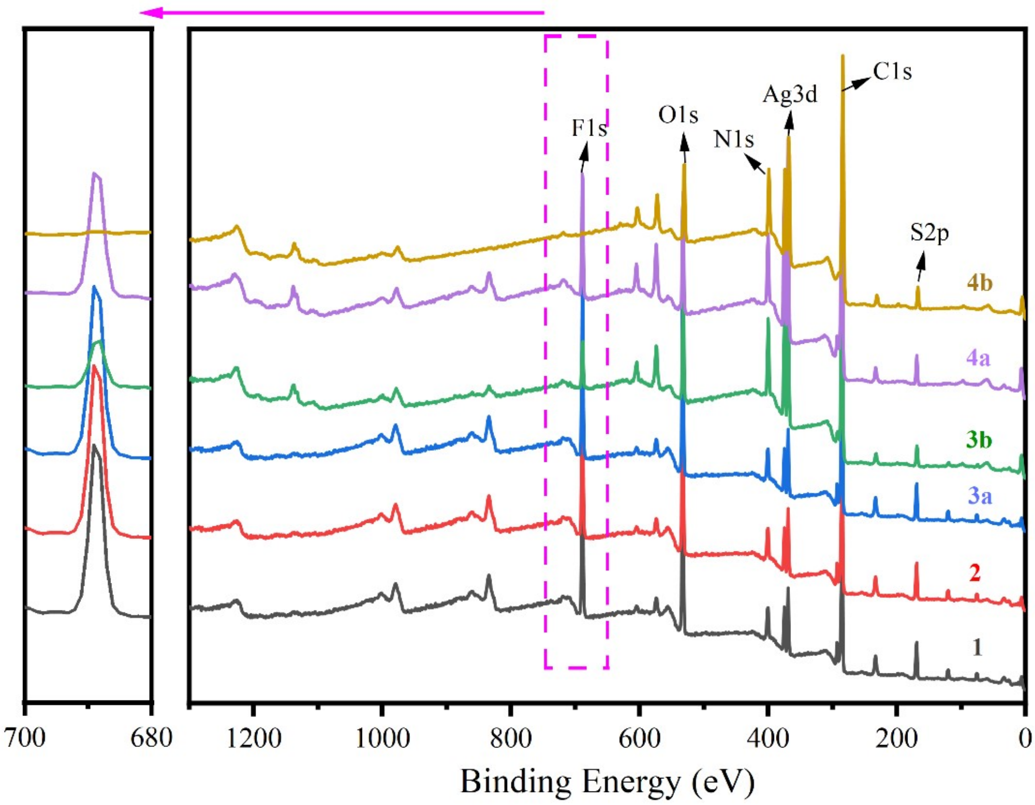

Figure S14. XPS spectra of **1–4b**.

7. Structures of Complexes 1–4b.

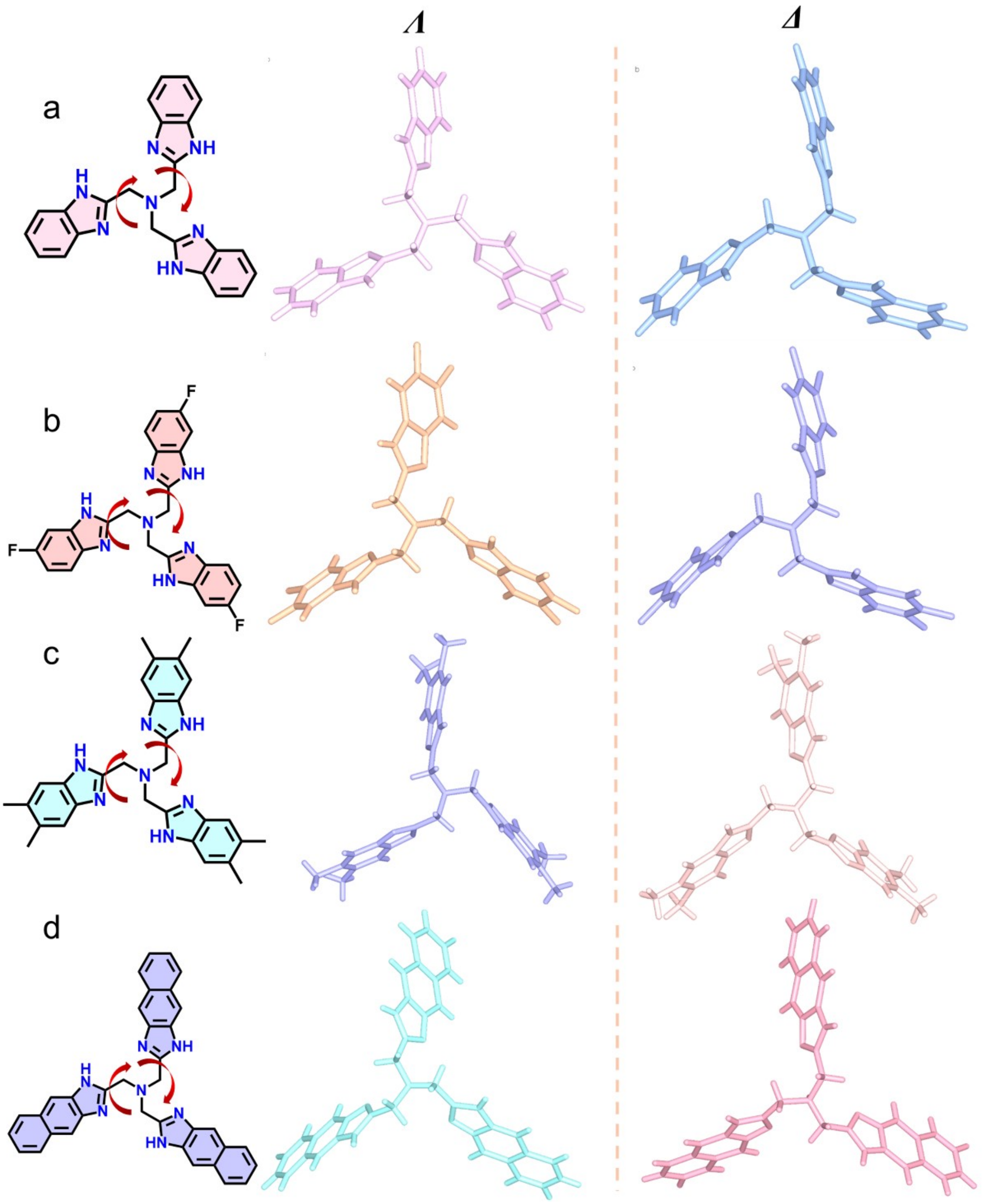

Figure S15. *Λ*- and *Δ*- conformations of ligands. (a) L^1. (b) L^2. (c) L^3. (d) L^4.

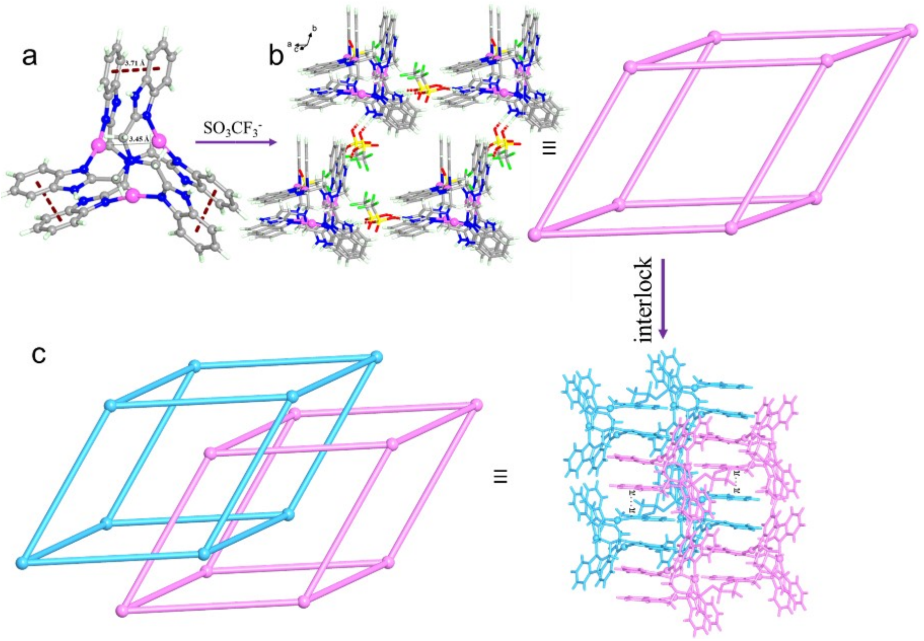

Figure S16. Crystal structure of **1**. (a) Trinuclear $[Ag_3L^1{}_2]^{3+}$ cation in **1**. (b) Single *α*-Po framework constructed by hydrogen bonds between OTf^- and $[Ag_3L^1{}_2]^{3+}$. (c) Dual interlocked *α*-Po network of **1**.

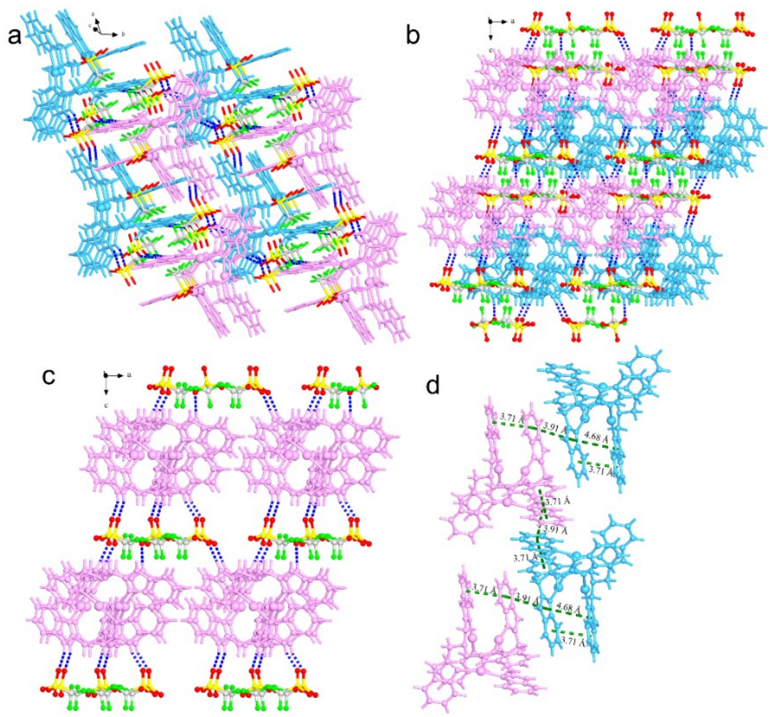

Figure S17. Crystal structure of **1**. (a) 2D supramolecular structure of **1** formed by hydrogen-bonded columns via inter-column $\pi\cdots\pi$ interactions. (b) Hydrogen-bonded column in **1**. (c) Single 2D supramolecular structure constructed by hydrogen bonds. (d) Inter-column $\pi\cdots\pi$ interactions in **1**.

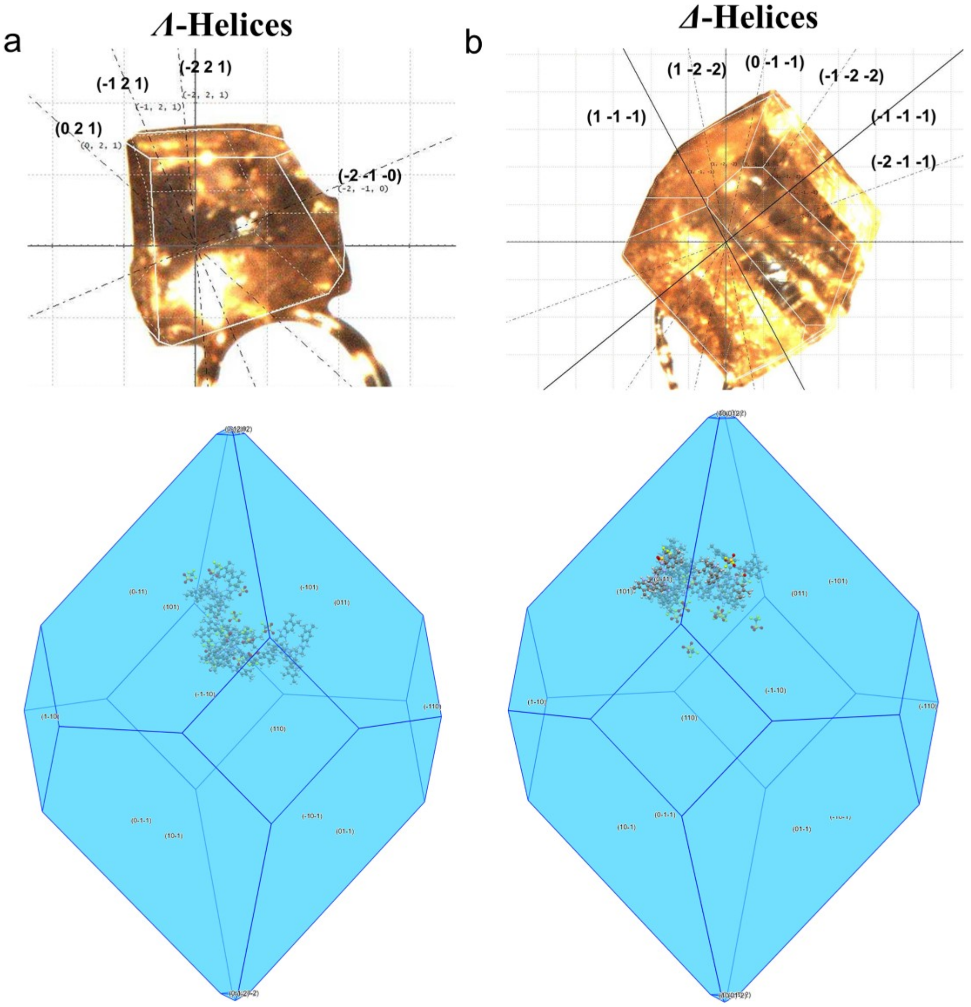

Figure S18. The Miller indices of the crystal faces and the predicted crystal shape of **3a** were determined using the BFDH method, with facet indexing performed by the Rigaku CryAlisPro software and shape prediction carried out by the Mercury software.

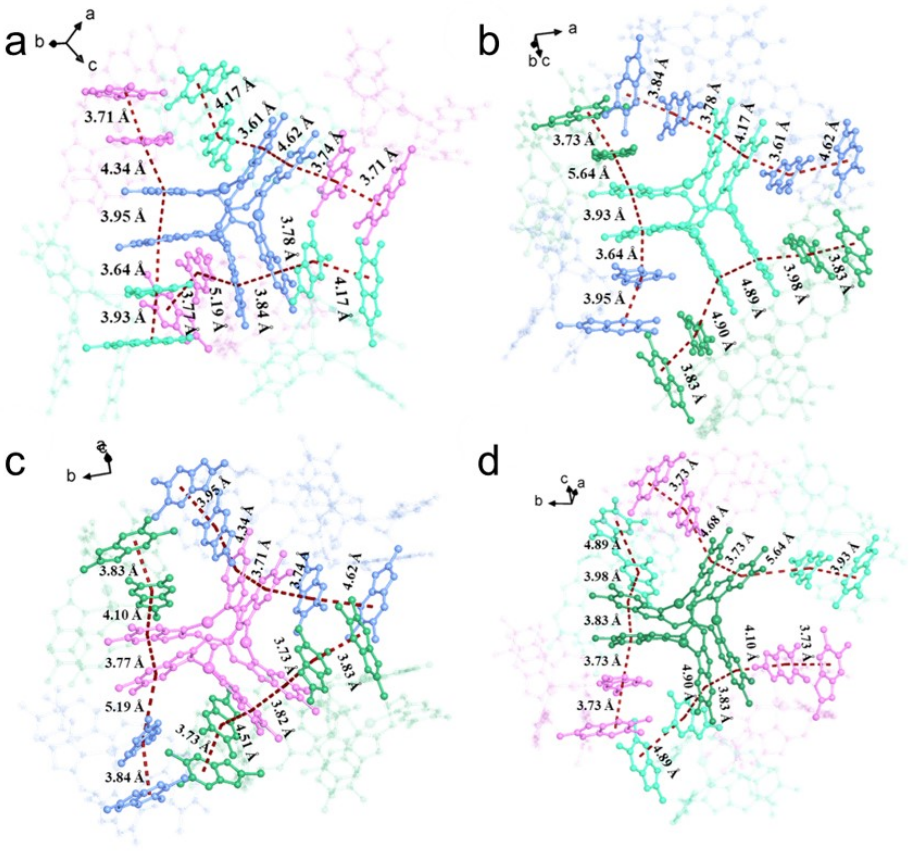

Figure S19. Intermolecular $\pi\cdots\pi$ interactions in **3a-*P*.** Each crystallographically different $[Ag_3L^3{}_2]^{3+}$ (shown in different color) in the asymmetric unit of **3a-*P*** associates with its six adjacent $[Ag_3L^3{}_2]^{3+}$ clusters forming the chiral 3D structure.

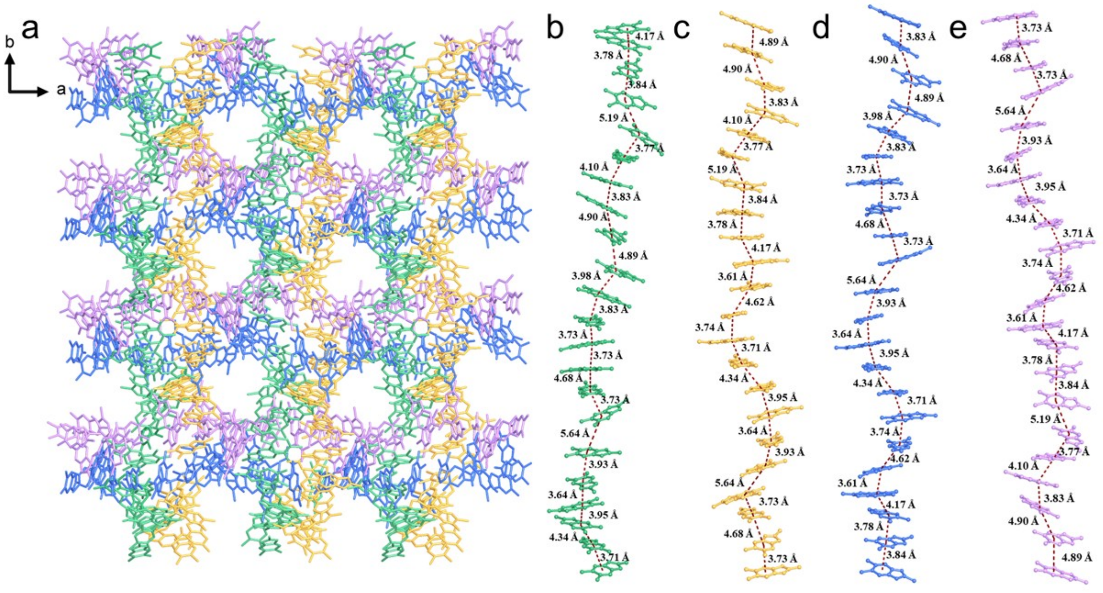

Figure S20. Four different π-stacked helical columns of $\Lambda\Lambda P$-$[Ag_3L^3{}_2]^{3+}$ and $\Lambda\Delta P$-$[Ag_3L^3{}_2]^{3+}$ of **3a-*P***.

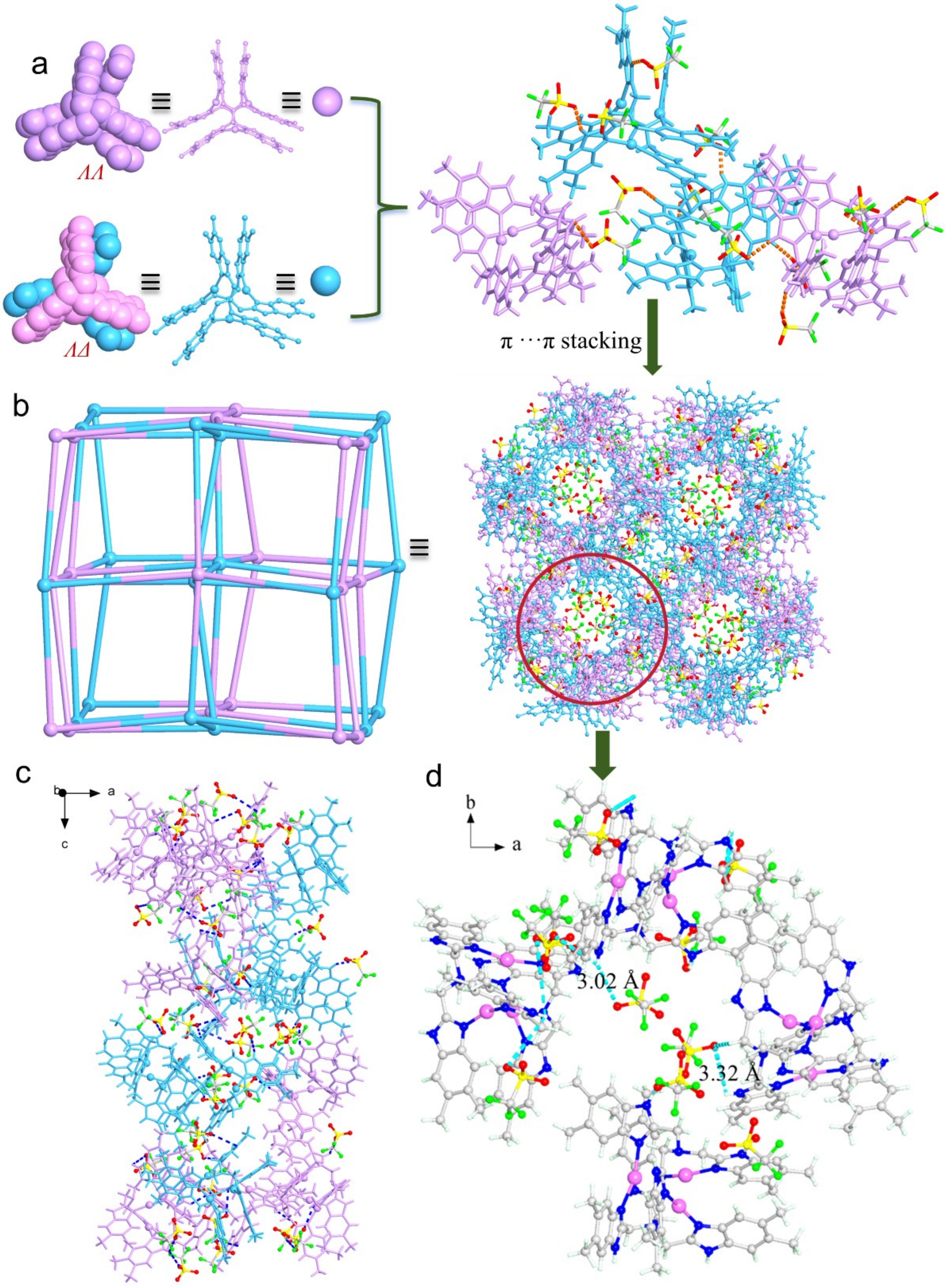

Figure S21. Crystal structure of **3a-*P***. (a) Asymmetric unit of **3a-*P*** and the structures of *ΛΛP*-$[Ag_3L^3{}_2]^{3+}$ and *ΛΔP*-$[Ag_3L^3{}_2]^{3+}$ in **3a-*P***. (b) Chiral 3D supramolecular structure of **3a-*P***. (c) Hydrogen-bonding interactions in the chiral 3D supramolecular structure of **3a-*P***. (d) View of OTf^- anions trapped in the channels of **3a-*P*** via NH···O hydrogen bonding.

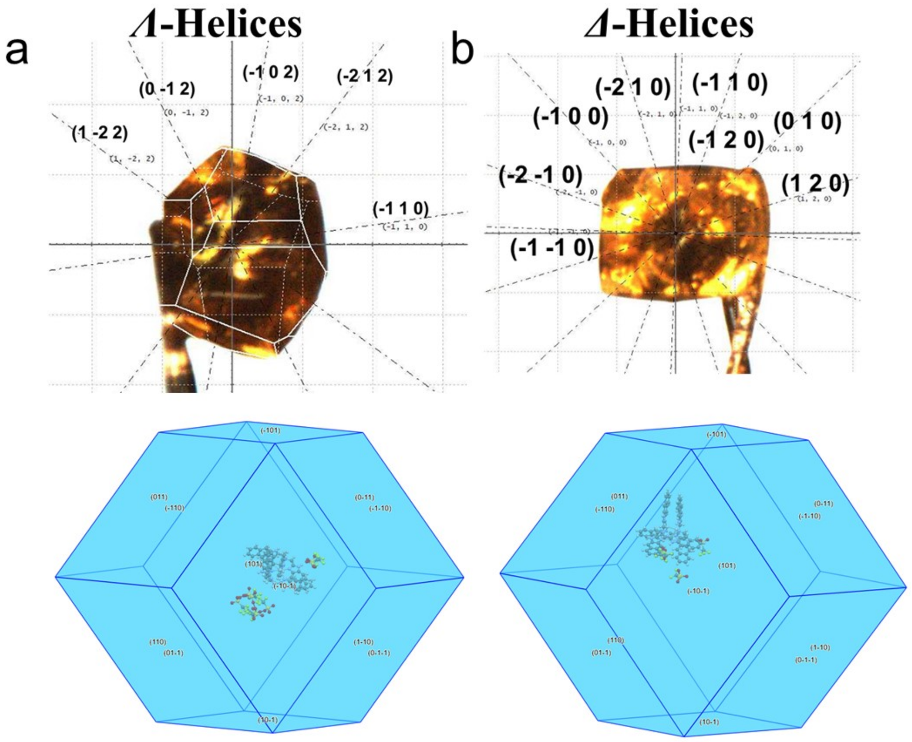

Figure S22. The Miller indices of the crystal faces and the predicted crystal shape of **4a** were determined using the BFDH method, with facet indexing performed by the Rigaku CryAlisPro software and shape prediction carried out by the Mercury software.

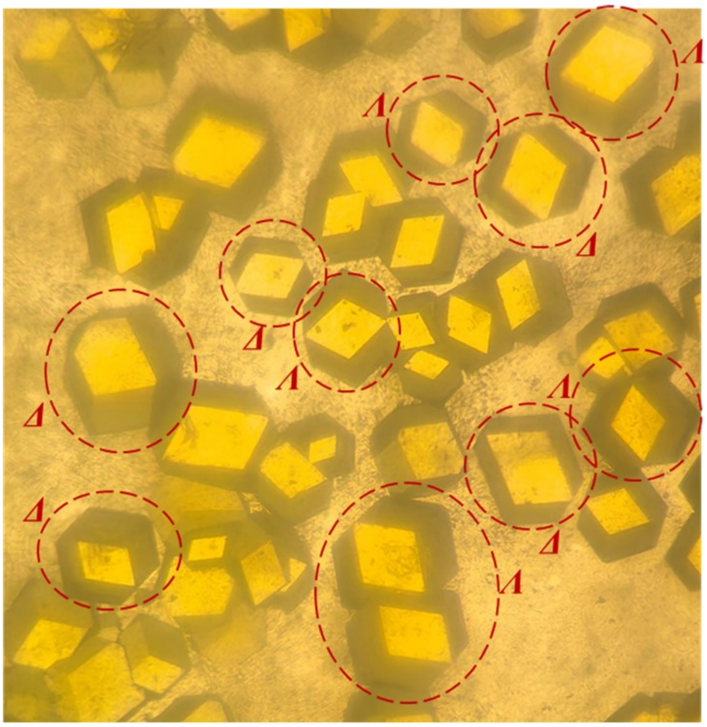

Figure S23. Photographic image of the crystals of **4a**.

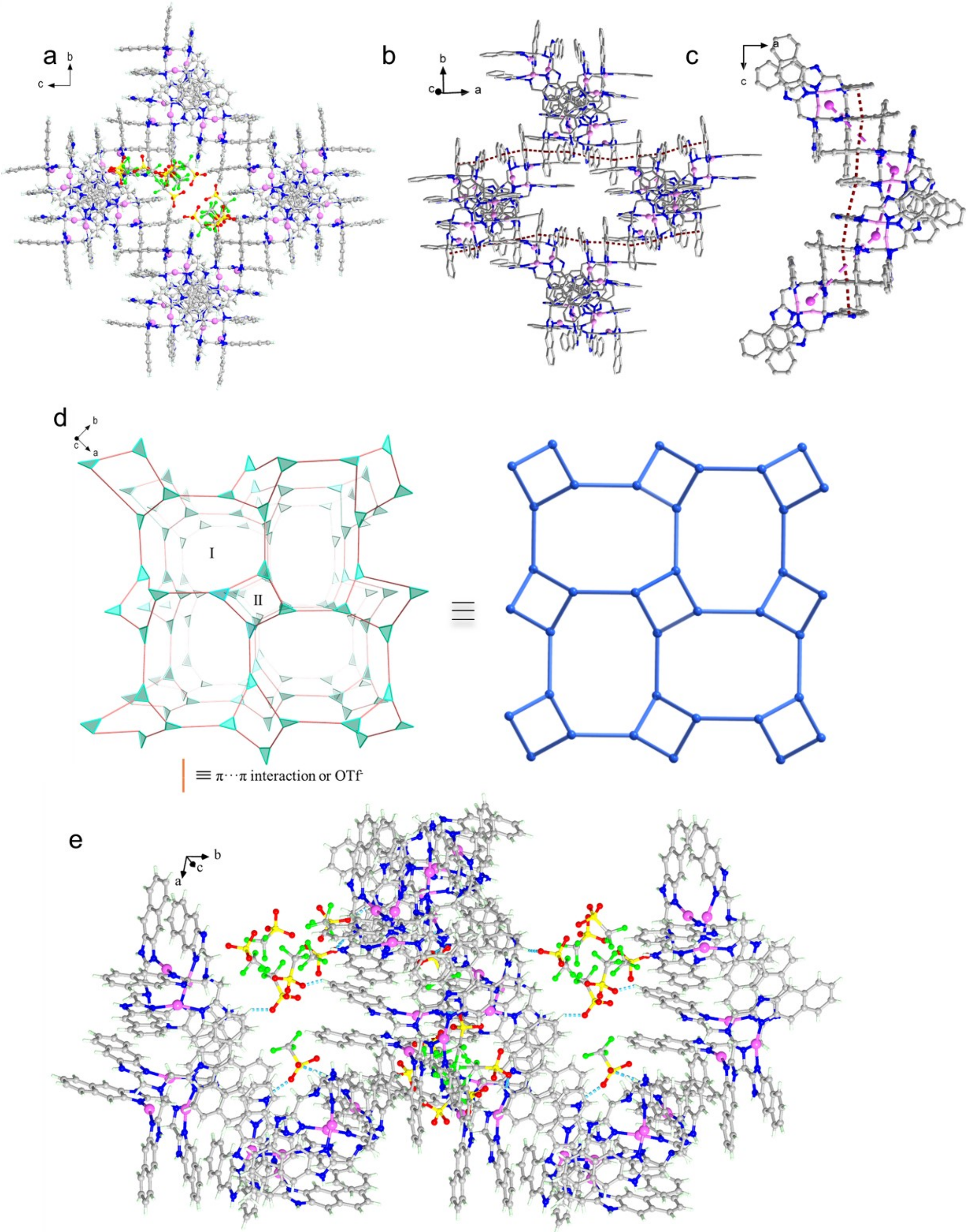

Figure S24. Crystal structure of **4a-*P***. (a) 3D chiral supramolecular framework structure of **4a-*P***. (b) Chiral ***srs*** network constructed by $\Lambda\Lambda P$-$[Ag_3L^4{}_2]^{3+}$ via $\pi\cdots\pi$ interactions. (c) Large helical nanotube formed by $\Lambda\Lambda P$-$[Ag_3L^4{}_2]^{3+}$ via $\pi\cdots\pi$ interactions. (d) Topology view of the chiral ***srs*** network of **4a-*P***. (e) Hydrogen-bonding interactions in the 3D chiral supramolecular structure of **4a-*P***.

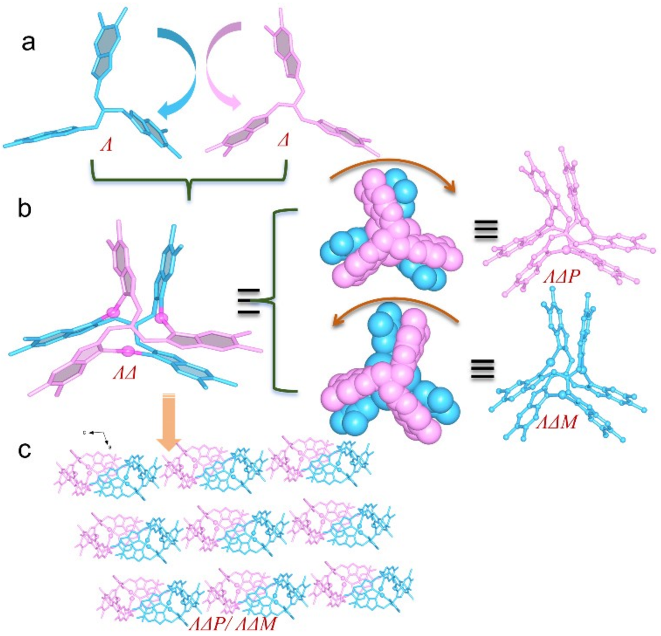

Figure S25. Racemic crystal structure of **3b.** (a) *Λ*- and *Δ*- conformations of ligand L^3. (b) *ΛΔP*- and *ΛΔM*-$[Ag_3L^3{}_2]^{3+}$ cation in **3b**. (c) Stacking of *ΛΔP*- and *ΛΔM*-$[Ag_3L^3{}_2]^{3+}$ in **3b**.

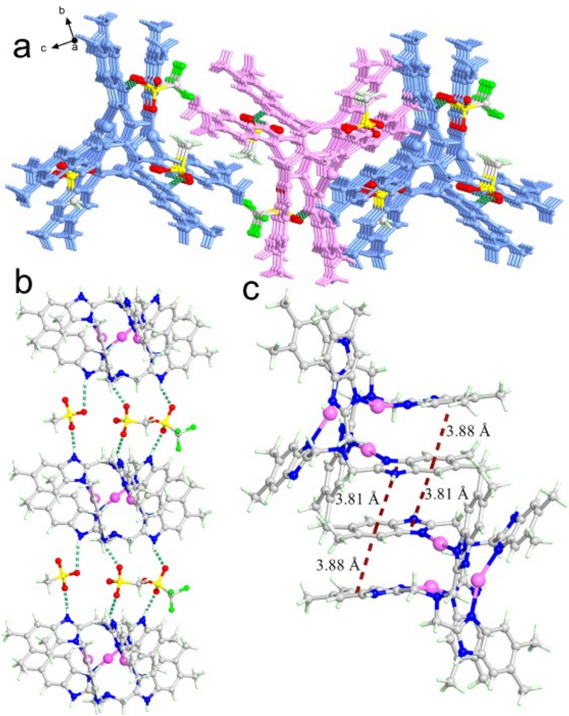

Figure S26. Hierarchical structure of **3b**. (a) 2D supramolecular structure of **3b** formed by hydrogen-bonded columns via inter-column $\pi\cdots\pi$ interactions. (b) Hydrogen-bonded column in **3b**. (c) Inter-column $\pi\cdots\pi$ interactions in **3b**.

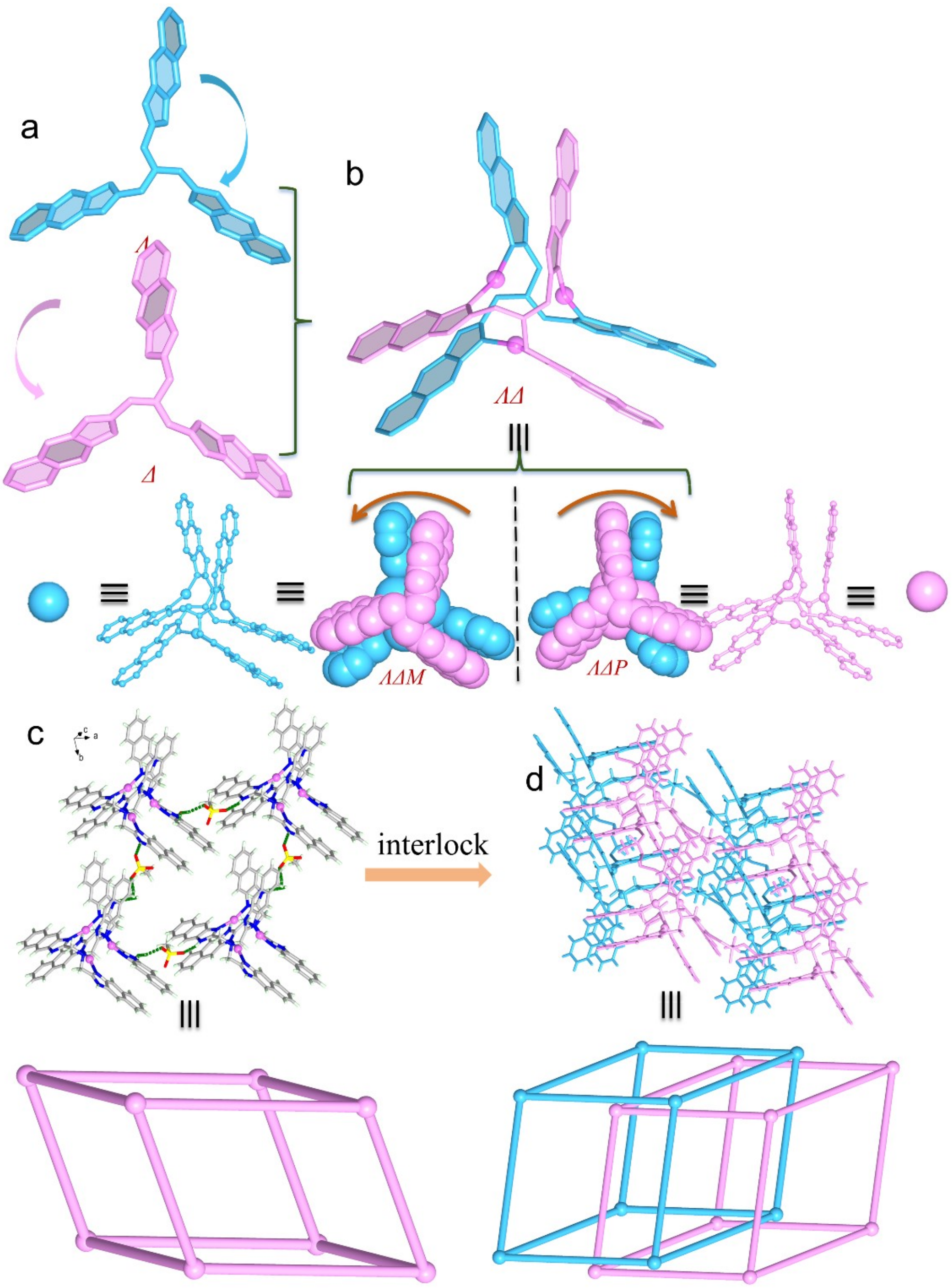

Figure S27. Racemic structure of **4b**. (a) *Λ*- and *Δ*-L^4 ligands. (b) *ΛΔP*- and *ΛΔM*-$[Ag_3L^4{}_2]^{3+}$ cluster in **4b**. (c) Single *α*-Po framework constructed by hydrogen bonding between $[(OMs^-)\cdot(H_2O)]$ and $[Ag_3L^4{}_2]^{3+}$. (d) Dual interlocked *α*-Po network of **4b**.

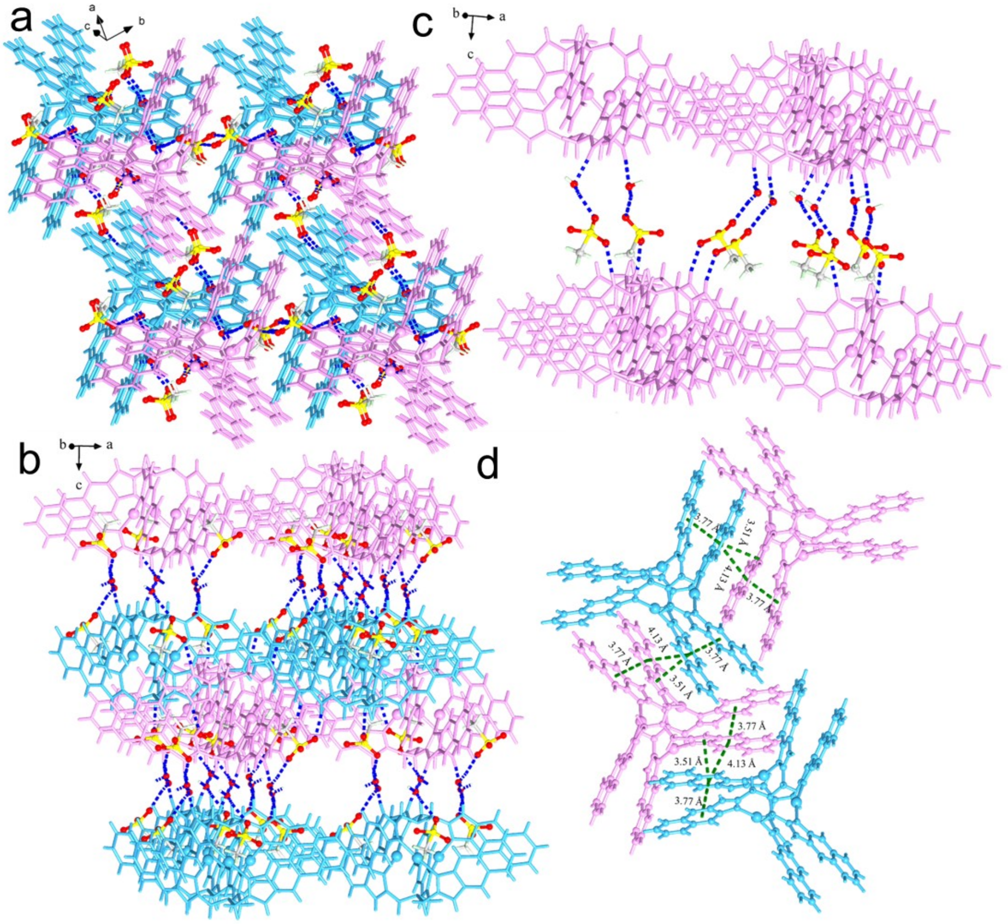

Figure S28. Crystal structure of **4b**. (a) 2D supramolecular structure of **4b** formed by hydrogen-bonded columns via inter-column $\pi\cdots\pi$ interactions. (b) Hydrogen-bonded column in **4b**. (c) Single 2D supramolecular structure constructed by hydrogen bonds. (d) Inter-column $\pi\cdots\pi$ interactions in **4b**.

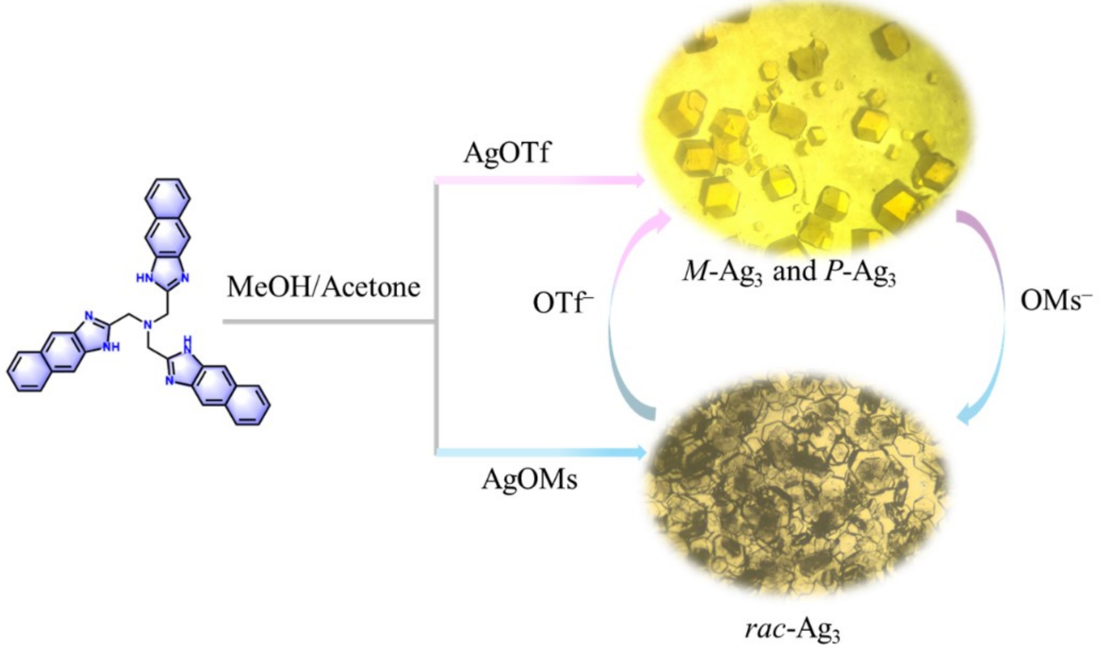

Figure S29. Interconversion between **4a** and **4b**.

8. ^{1}H NMR and SHG Responses of 3a, 3b, 4a, and 4b.

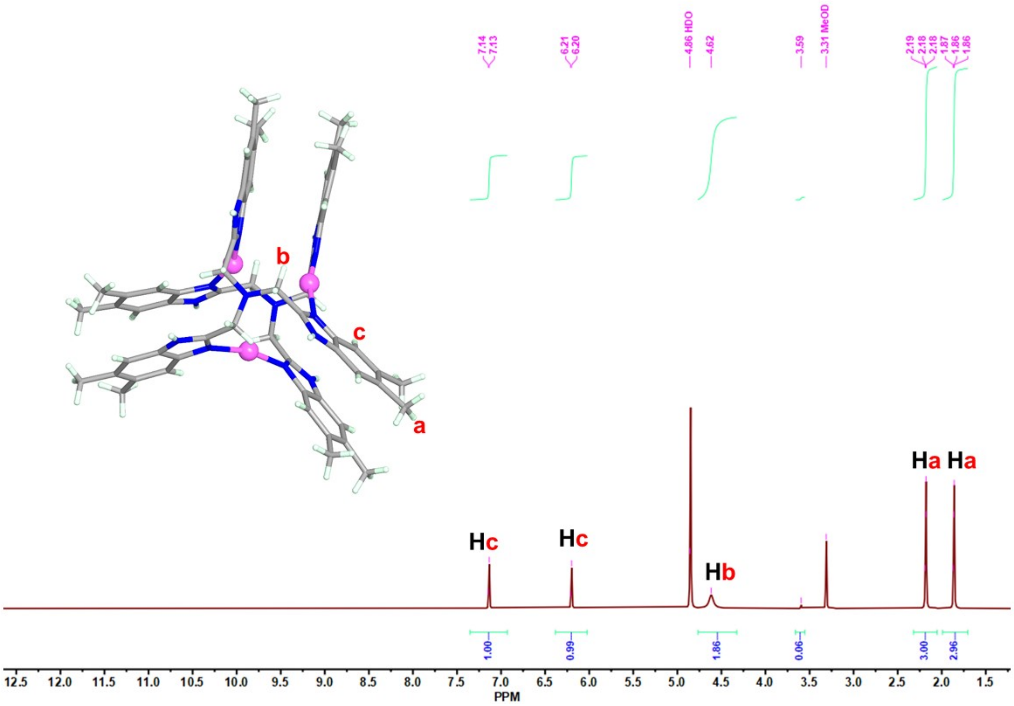

Figure S30. The ^{1}H NMR spectrum of **3a** crystals dissolved in deuterated methanol.

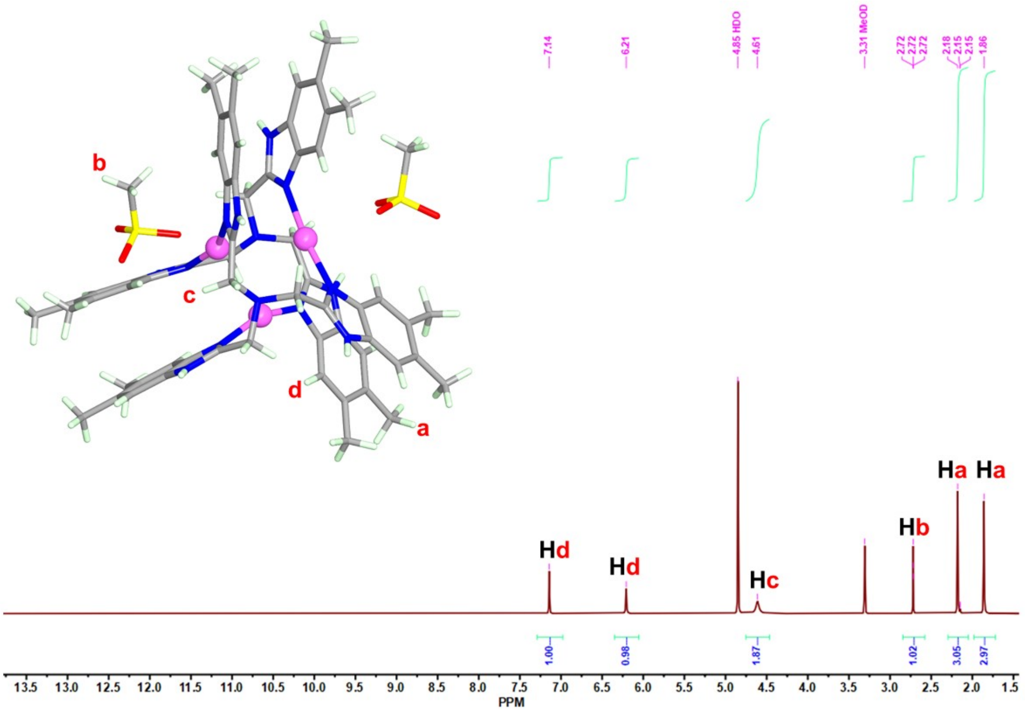

Figure S31. The ^{1}H NMR spectrum of **3b** crystals dissolved in deuterated methanol.

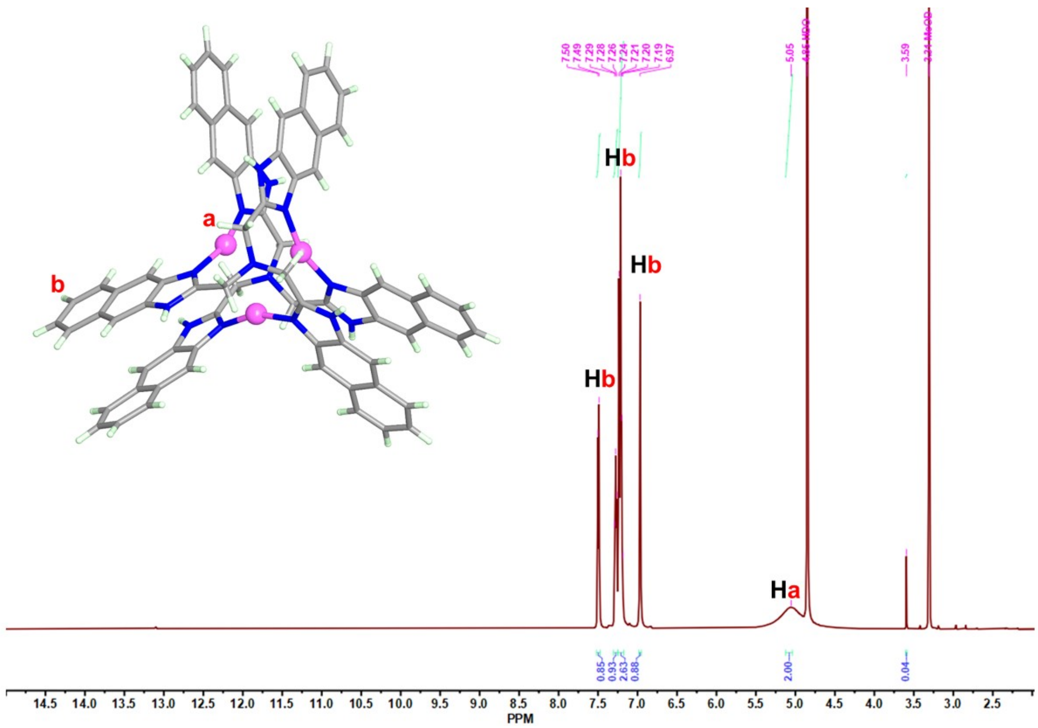

Figure S32. The ^{1}H NMR spectrum of **4a** crystals dissolved in deuterated methanol.

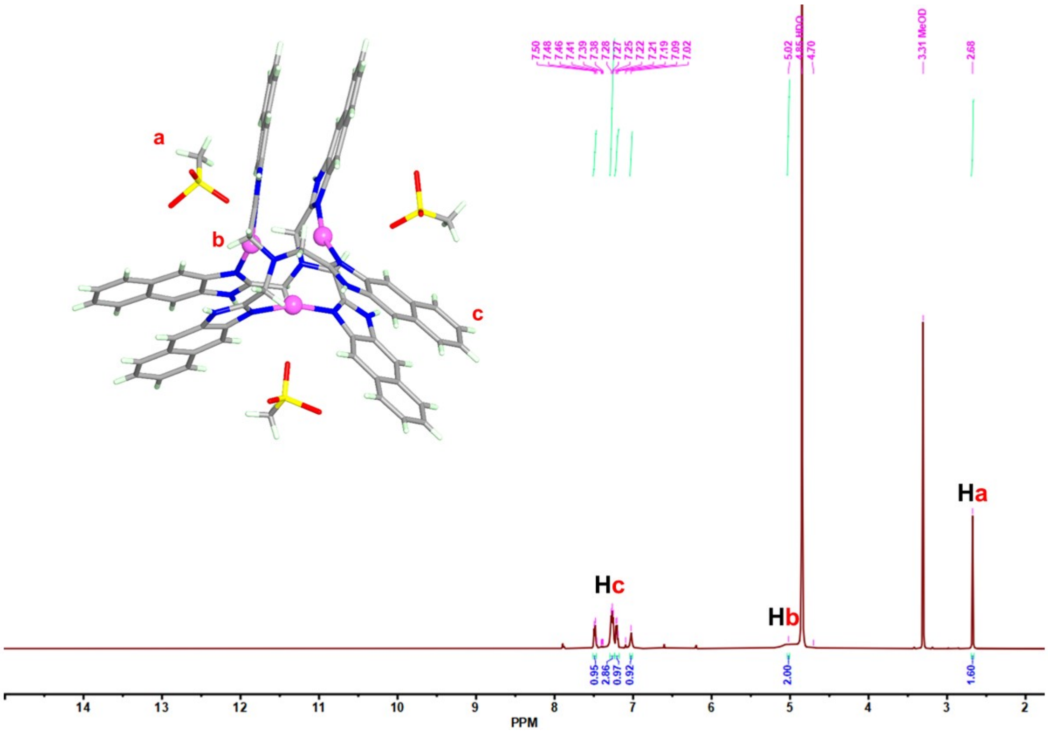

Figure S33. The ^{1}H NMR spectrum of **4b** crystals dissolved in deuterated methanol.

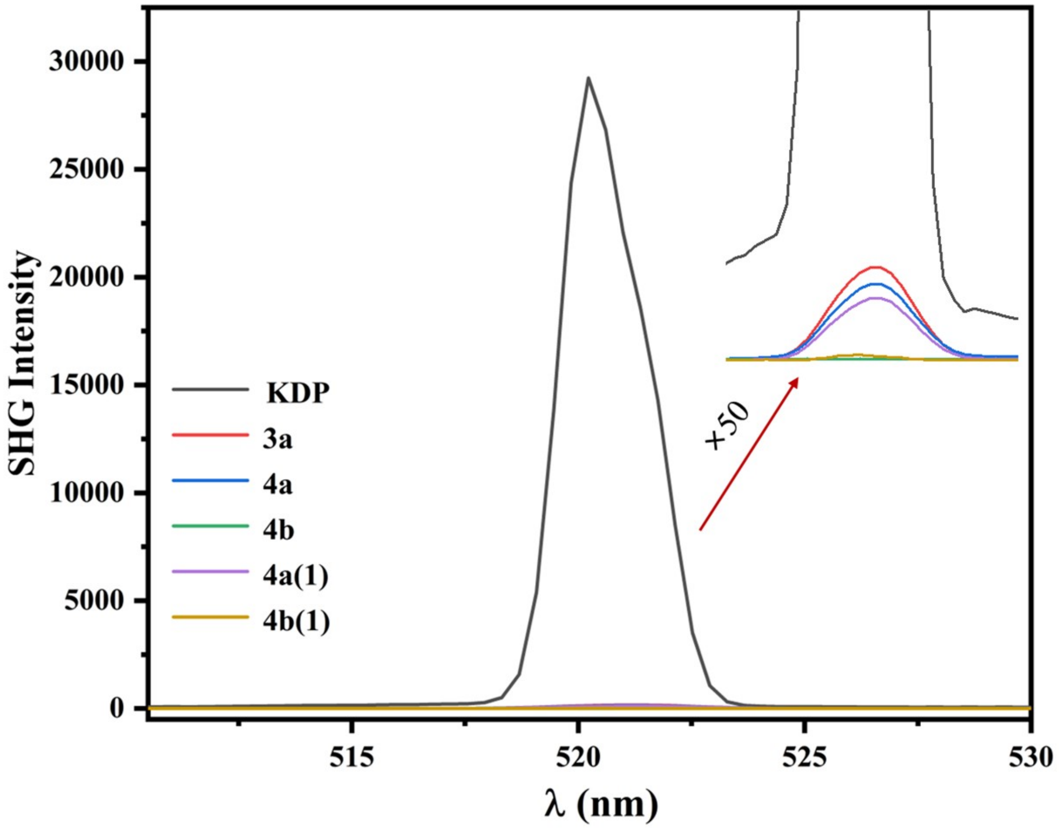

Figure S34. SHG signals of crystals of compounds **3a**, **4a**, and **4b** were measured using KH_2PO_4 (KDP) as the reference. (**4a(1)** was obtained by recrystallization of **4b** in a NaOTf-containing solution, and **4b(1)** was obtained by recrystallization of **4a(1)** in a NaOMs-containing solution.)

9. Supplementary References

S1. V.O. Rodionov, S.I. Presolski, S. Gardinier, Y. H. Lim, M. G. Finn, *J Am Chem Soc*. 2007, **129**, 12696–1270.

S2. G.M. Sheldrick, *Acta Crystallogr A Found Adv*. 2015, **71**, 3–8.
